# The Antioxidant and HDAC-Inhibitor α-Lipoic Acid Is Synergistic with Exemestane in Estrogen Receptor-Positive Breast Cancer Cells

**DOI:** 10.3390/ijms25158455

**Published:** 2024-08-02

**Authors:** Laura S. Pradel, Yu-Lin Ho, Holger Gohlke, Matthias U. Kassack

**Affiliations:** 1 Institute for Pharmaceutical and Medicinal Chemistry, Heinrich Heine University Düsseldorf, 40225 Düsseldorf, Germany; laura.pradel@hhu.de (L.S.P.); yu-lin.ho@hhu.de (Y.-L.H.); gohlke@uni-duesseldorf.de (H.G.); 2 Institute of Bio- and Geosciences (IBG-4: Bioinformatics), Forschungszentrum Jülich, 52425 Jülich, Germany

**Keywords:** breast cancer, estrogen receptor, endocrine therapy, histone deacetylase inhibitor, aromatase inhibitor, alpha-lipoic acid, exemestane, combination therapy, synergy

## Abstract

Anti-estrogenic therapy is established in the management of estrogen receptor (ER)-positive breast cancer. However, to overcome resistance and improve therapeutic outcome, novel strategies are needed such as targeting widely recognized aberrant epigenetics. The study aims to investigate the combination of the aromatase inhibitor exemestane and the histone deacetylase (HDAC) inhibitor and antioxidant α-lipoic acid in ER-positive breast cancer cells. First, the enantiomers and the racemic mixture of α-lipoic acid, and *rac*-dihydro-lipoic acid were investigated for HDAC inhibition. We found HDAC inhibitory activity in the 1–3-digit micromolar range with a preference for HDAC6. *Rac*-dihydro-lipoic acid is slightly more potent than *rac*-α-lipoic acid. The antiproliferative IC_50_ value of α-lipoic acid is in the 3-digit micromolar range. Notably, the combination of exemestane and α-lipoic acid resulted in synergistic behavior under various incubation times (24 h to 10 d) and readouts (MTT, live-cell fluorescence microscopy, caspase activation) analyzed by the Chou–Talalay method. α-lipoic acid increases mitochondrial fusion and the expression of apoptosis-related proteins p21, APAF-1, BIM, FOXO1, and decreases expression of anti-apoptotic proteins survivin, BCL-2, and c-myc. In conclusion, combining exemestane with α-lipoic acid is a promising novel treatment option for ER-positive breast cancer.

## 1. Introduction

The rate of new breast cancer cases grows every year by 0.5%, demonstrating the importance of improving treatment with repurposed drugs and novel targeted strategies [1,2]. Around 80% of all breast cancers are estrogen receptor (ER)-positive. These patients receive adjuvant endocrine therapy in addition to surgery and/or radiation therapy [3]. Endocrine therapy comprises estrogen receptor modulators (SERMs) such as tamoxifen and raloxifen and aromatase inhibitors such as competitive letrozole or irreversible steroidal inhibitor exemestane [4]. Goss et al. reported that exemestane reduced the number of invasive breast cancers compared to placebo in postmenopausal women with fewer side effects than the above-mentioned SERMs [5]. Although endocrine therapy is among the most effective treatment strategies in ER-positive breast cancer, resistance against endocrine therapy emerges with cancer progression [6]. Novel therapeutic strategies are thus urgently needed. Molecular and genetic characterization of cancers has been summarized by Hanahan and Weinberg in their seminal paper about the hallmarks of cancer [7] and has recently been updated by Hanahan [8]. Non-mutational epigenetic reprogramming is one of the newly added features of cancers, and treatment with histone deacetylase inhibitors (HDACi) is one of the strategies to target aberrant epigenetics in cancers [9]. Several HDACi are approved for the treatment of hematological malignancies. In recent studies, we have demonstrated that class-selective HDACi are synergistic with anticancer drugs of different mechanisms of action. The novel class IIa HDACi YAK540 is synergistic with bortezomib in leukemia cell lines [10]. Class I selective HDACi and class I/IIb HDACi revert chemoresistance in cellular models of platinum resistance in head–neck and ovarian cancer cell lines [11]. In breast cancer, several preclinical and clinical studies have shown benefits from the addition of HDACi to established therapies [12,13]. A phase II study has revealed that the HDACi vorinostat was able to reverse hormone-resistance in breast cancer [14]. Another phase II trial studying the combination of exemestane and the class I HDACi entinostat showed benefits for the combination compared to exemestane single treatment [15]. A subsequent phase III trial in advanced hormone receptor-positive breast cancer with exemestane and entinostat could not confirm improved survival compared to exemestane alone [16]. However, in a Chinese phase III study in advanced hormone receptor-positive breast cancer, the combination of exemestane and entinostat significantly improved progression-free survival [17], which was also found in a phase III trial for the combination of exemestane and tucidinostat [18]. Furthermore, dietary compounds may serve as HDACi and contribute to epigenetic modulation, such as butyrate, diallyl disulfide, sulforaphane, and α-lipoic acid [19,20]. Recently, Lechner et al. revealed histone deacetylases as targets of (*R*)-α-lipoic acid [21]. They report that the reduced forms of racemic or (*R*)-α-lipoic acid, i.e., *rac*-dihydro-lipoic acid, are the most potent HDACi in the α-lipoic acid family, particularly at HDAC6 (IC_50_ around 1 µM). Further, they found no HDAC inhibitory activity for (*S*)-α-lipoic acid up to 500 µM. Based on these data, Watson et al. described a crystal structure of HDAC6 with dihydro-lipoic acid [22]. α-lipoic acid is known as an essential cofactor of several mitochondrial enzyme complexes involved in energy metabolism. In addition, it is a strong antioxidant and reactive oxygen species (ROS) scavenger and is used as a food supplement and approved drug for various purposes including the treatment of diabetic polyneuropathy, liver diseases, and ROS-associated diseases [23]. In addition, α-lipoic acid was reported to have various anticancer effects via its antioxidative nature, induction of cell cycle arrest and apoptosis, synergy with radiotherapy, and inhibition of IGF1-R maturation [23,24,25,26]. The encouraging effects of HDACi and in particular the pleiotropic anticancer effects of α-lipoic acid prompted us to investigate the combination of the irreversible aromatase inhibitor exemestane with the antioxidant and HDACi α-lipoic acid in the ER-positive breast cancer cell lines MCF-7 and T47D. First, we investigated the effects of the enantiomers and the racemic mixture of α-lipoic acid on HDAC inhibition and the proliferation of ER-positive breast cancer cell lines. The main aim of this study was, however, to explore a possible synergy between exemestane and α-lipoic acid under various incubation conditions. Surprisingly, the S-enantiomer emerged as HDACi, particularly on HDAC6, with only slightly reduced potency compared to (*R*)-α-lipoic acid, and displayed similar cytotoxicity. Notably, the combination of exemestane and α-lipoic acid showed synergistic effects in cytotoxicity and caspase activation as shown by Chou–Talalay analysis [27,28], and led to an increase in p21 and apoptotic proteins. In summary, our study suggests the combination of exemestane and α-lipoic acid as a promising novel treatment option for ER-positive breast cancer.

## 2. Results

### 2.1. HDAC-Inhibitory Effects of (R)-, (S)-, Rac-α-Lipoic Acid, and Rac-Dihydro-Lipoic Acid

Prior to the publication of Lechner et al. [21], we had already started to investigate the enantiomers and the racemic mixture of α-lipoic acid as well as the reduced racemic mixture, i.e., rac-dihydro-lipoic acid at selected HDAC isoforms presenting the major HDAC classes. HDAC2 and 8 are representatives of class I, HDAC4 of class IIa, and HDAC6 of class IIb. All of the α-lipoic acid forms investigated by us were obtained from Biosynth, except for (*R*)-α-lipoic acid, which was obtained from MedChemExpress, providing certificates of analysis for each batch used by us. In addition, we performed high-resolution mass spectrometry and ^1^H-NMR analysis of the α-lipoic acid derivates (Figure S1a and S1b), demonstrating the identity and purity, except determination of the absolute stereochemistry. In contrast to results from Lechner et al., (*S*)-α-lipoic acid was only a slightly less potent HDACi than (*R*)-α-lipoic acid at all investigated HDAC enzymes with particular potency at HDAC6, where it obtained an IC_50_ value of 21.3 µM [21]. IC_50_ values of all HDAC enzyme assays are presented in Table 1, together with reference HDACi. (*S*)-α-lipoic acid is at least 14-fold selective for HDAC6 and can thus be classified as a class IIb-preferential HDACi. The same holds true for (*R*)-α-lipoic acid, racemic α-lipoic acid, and *rac*-dihydro-lipoic acid which all show HDAC6 preference (10-fold, 13-fold, 8-fold, respectively, Table 1). Further, the potency of (*R*)-α-lipoic acid, (*S*)-α-lipoic acid, *rac*-α-lipoic acid, and *rac*-dihydro-lipoic acid is approximately the same at all HDAC enzymes investigated. *Rac*-dihydro-lipoic acid is only slightly more potent at HDAC2 (2.1-fold), HDAC6 (2.2-fold), and HDAC8 (2.9-fold) than (*R*)-α-lipoic acid, whereas Lechner et al. reported a somewhat superior potency of *rac*-dihydro-lipoic acid compared to α-lipoic acid [21].

**Figure 1:**
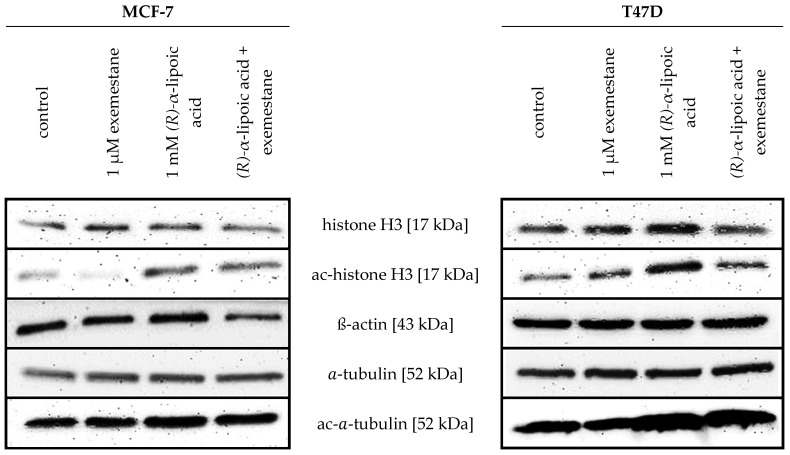
Effect of (*R*)-α-lipoic acid on acetylation level of a-tubulin and histone H3. Representative immunoblot analysis of histone H3, ac-histone H3, a-tubulin, and ac-a-tubulin in ER+ breast cancer cell lines MCF-7 and T47D. Cells were treated with 1 mM (R)-α-lipoic acid, 1 µM exemestane, as well as a combination of 1 mM (R)-α-lipoic acid and 1 µM exemestane for 48 h. ß-actin served as loading control. Uncropped and labeled immunoblots are presented in Figure S2.

In addition to enzyme HDAC assays, we wanted to demonstrate that α-lipoic acid is also inhibiting HDACs in a cellular environment. We thus performed cellular HDAC assays using the class I and IIb-selective cell-permeable HDAC substrate Boc-Lys-AC-AMC in T47D cells (Table 1). In addition, we investigated the acetylation of histone and alpha-tubulin in MCF-7 and T47D cells by western blotting (Figure 1). Again, (*R*)-α-lipoic acid, (*S*)-α-lipoic acid, and the racemic mixture show similar potencies in the cellular HDAC assays (Table 1). Assuming that α-lipoic acid undergoes reduction to dihydro-lipoic acid in the reductive cellular environment, the loss of HDAC inhibitory activity in the cellular environment compared to enzymatic HDAC2 inhibition is similar for α-lipoic acid as it is for the reference compound vorinostat (Table 1). Vorinostat had an IC_50_ at HDAC2 of 0.12 µM and an IC_50_ in the cellular HDAC assay in T47D cells of 0.79 µM, which is comparable to the IC_50_ of 0.88 µM in the squamous carcinoma cell line Cal27 previously reported by us [29]. Although IC_50_ values of α-lipoic acid in the cellular HDAC assays are between 906 and 1186 µM, a cellular effect can be assumed at much lower concentrations due to the low IC_50_ of α-lipoic acid at HDAC6 (6.48–21.3 µM). This was confirmed by western blotting showing increased acetylation of histone and of alpha-tubulin after treatment with (*R*)-α-lipoic acid or a combination of (*R*)-α-lipoic acid and exemestane but not with exemestane only (Figure 1).

### 2.2. Cytotoxic Effects of α-Lipoic Acid and Exemestane

First, the physiological R-enantiomers of α-lipoic acid and exemestane were tested in a 72 h MTT assay for their cytotoxic potency in ER-positive MCF-7 and T47D and as control in the triple-negative breast cancer cell line MDA-MB-231. IC_50_ values are shown in Table 2. Exemestane is slightly more potent in MCF-7 than in T47D cells but clearly less potent in MDA-MB-231 cells (3-fold compared to MCF-7; 1.8-fold compared to T47D). Because MDA-MB-231 is ER-negative, this result is plausible. Compared to nanomolar inhibition of the aromatase, the IC_50_ of exemestane inhibiting cell proliferation is rather high, but in accordance with the literature data reporting an IC_50_ of 30 µM for exemestane in MCF-7 cells [30]. (*R*)-α-lipoic acid showed an IC_50_ value of around slightly above 1 mM in MCF-7 and T47D cells and slightly below 1 mM in MDA-MB-231. This is in agreement with Farhat et al. who have shown that α-lipoic acid exerts a cytotoxic effect in breast cancer cells only at higher concentrations and independently from the hormone receptor status [31].

Since the IC_50_ values of (*R*)-α-lipoic acid were around 1 mM, we next extended the incubation time to 120 h and investigated the enantiomers and the racemic mixture of α-lipoic acid as well as exemestane. IC_50_ values are shown in Table 3. Prolongation of the incubation time increased the potency of exemestane by a factor of 1.8 in ER-positive and 1.7 in triple-negative breast cancer cells. For (*R*)-α-lipoic acid, the increase in potency was more pronounced: 2.7/2.6-fold in MCF-7/T47D and 3.9-fold in MDA-MB-231 cells. The R-enantiomer, the S-enantiomer, and the racemic mixture showed a similar cytotoxicity in the respective cell lines, which is in agreement with our results of the HDAC enzyme assays (Table 1). Interestingly, the cytotoxicity of all α-lipoic acid derivatives is around 2-fold increased in the triple negative breast cancer cell line MDA-MB-231 cells compared to the ER-positive cancer cells (Table 3).

### 2.3. Combination of R-α-Lipoic Acid and Exemestane and Synergy Analysis

**Figure 2:**
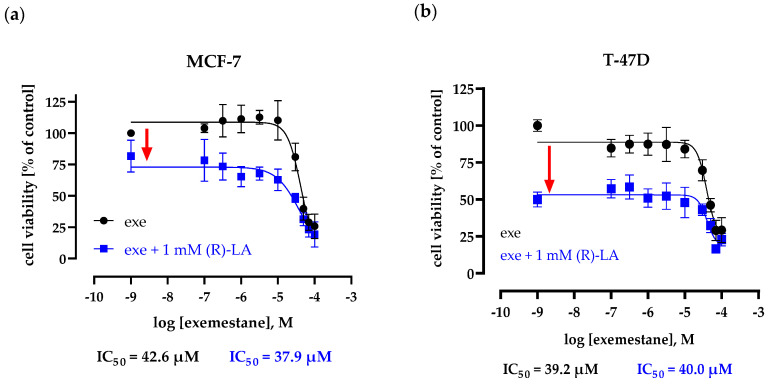
Concentration–effect curve of exemestane in the absence and presence of 1 mM (*R*)-α-lipoic acid. Cell viability is measured using MTT assay after 72 h incubation in MCF-7 (**a**) and T47D (**b**). Data are the mean of at least two different experiments ± SD, each carried out in triplicates. Exe = exemestane. (*R*)-LA = (*R*)-α-lipoic acid.

Next, the effect of the physiological α-lipoic acid, i.e., the R-enantiomer, on exemestane and a possible synergistic behavior were analyzed in the ER-positive breast cancer cell lines. First, concentration–effect curves of exemestane in the absence and presence of 1 mM (*R*)-α-lipoic acid, approximately corresponding to its IC_50_ values, were monitored in MCF-7 and T47D cells (Figure 2). In MCF-7 cells, increasing concentrations of exemestane up to 10 µM in the absence of α-lipoic acid led to a slight increase in proliferation, possibly due to a weak agonistic activity of exemestane at the ER receptors previously described in the literature [32]. A quantity of 1 mM (*R*)-α-lipoic acid had no significant effect on the IC_50_ of exemestane but decreased cell viability by approximately 35% in both cell lines (Figure 2).

**Figure 3:**
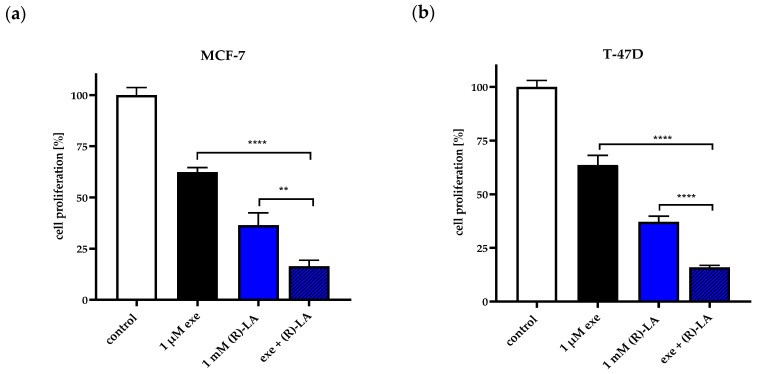
Cell proliferation of MCF-7 (a) and T47D cells (b) measured via Hoechst 33342 staining. Hoechst 33342-stained nuclei were counted after 72 h incubation with 1 µM exemestane or/and 1 mM (*R*)-α-lipoic acid. Shown is one representative experiment out of a series of at least three experiments each performed with 3 replicates. Data are mean ± SD. Statistical comparison was performed using a t-test. Levels of significance: ** (*p* ≤ 0.01); **** (*p* ≤ 0.0001). Exe = exemestane. (*R*)-LA = (*R*)-α-lipoic acid.

In addition to the MTT assay monitoring viable cells (Figure 2), cell proliferation of ER-positive cells in the presence of exemestane and (*R*)-α-lipoic acid was determined by counting Hoechst 33342-stained nuclei via fluorescence microscopy (Figure 3). The 1 µM exemestane decreases the number of nuclei by around 37% in both cell lines which is clearly more than the effect of the 1 µM exemestane in the MTT assay (Figure 2). Similarly, the effect of 1 mM (*R*)-α-lipoic acid is more pronounced in nuclear count readout than with the MTT assay. Most interestingly, the effect of the combination of 1 µM exemestane and 1 mM (*R*)-α-lipoic acid is even stronger as it leaves only around 16% of nuclei compared to control (Figure 3). In contrast, the combination of 1 µM exemestane and 1 mM (*R*)-α-lipoic acid gave no additional effect (T47D) or only a slight effect (MCF-7) compared to 1 mM (*R*)-α-lipoic acid alone in the MTT assay (Figure 2).

**Figure 4:**
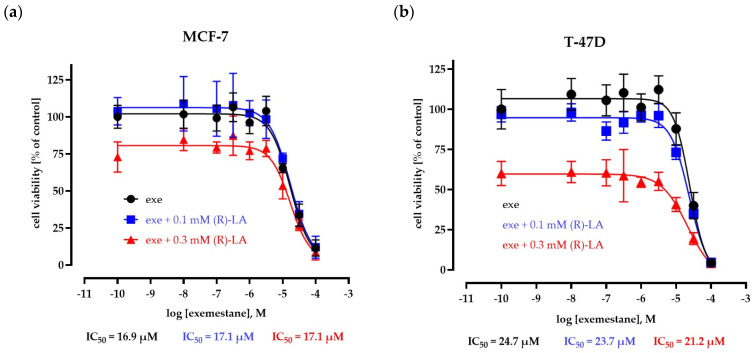
Concentration–effect curves of exemestane in the absence and presence of 100 µM and 300 (*R*)-α-lipoic acid. Cell viability is measured using MTT assay after 120 h incubation in MCF-7 (**a**) and T47D cells (**b**). Data are mean of the of two different experiments ± SD, each carried out in triplicates. Exe = exemestane. (*R*)-LA = (*R*)-α-lipoic acid.

**Figure 5:**
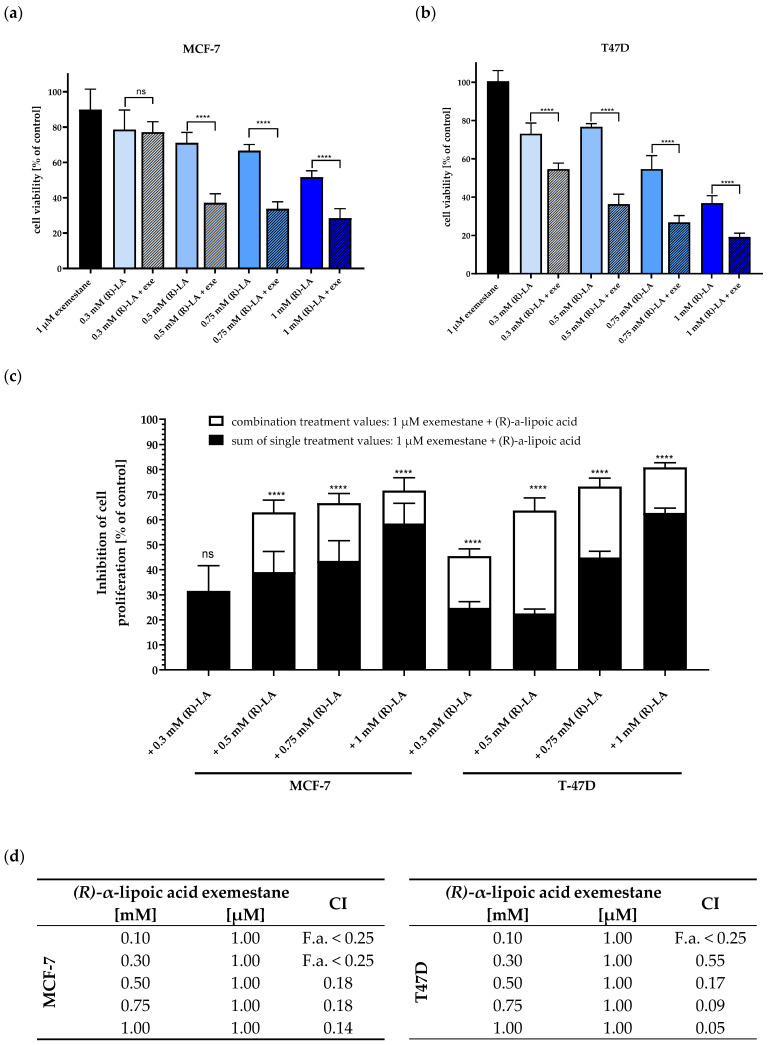
Combination treatment of 1 µM exemestane and (*R*)-α-lipoic acid. (**a**,**b**) show cell viability of MCF-7 (**a**) and T-47D (**b**) normalized to untreated control estimated via MTT assay after 120 h incubation. (**c**) Inhibition of cell viability based on data from (**a**,**b**): black bars represent the effects of the sum of single treatments, white bars illustrate the effect of the combination treatments. Data shown are mean ± SD of at least two independent experiments, carried out in 6 replicates. Statistical analysis was performed using a *t*-test. Levels of significance: **** (*p* < 0.0001). (**d**) Combination index (CI) values from the Chou–Talalay analysis of data are shown in Figure 4 and Figure 5a,b [27,28]. Fractions affected were between 0.25 and 0.8. Exe = exemestane. (*R*)-LA = (*R*)-a-lipoic acid. ns = not significant.

Since we observed these differences between MTT assay and Hoechst 33342 nuclear stain assay both performed for 72 h, we next investigated both compounds in MTT assays with an extended incubation time of 120 h (Figure 4 and Figure 5). Figure 4 shows concentration–effect curves of exemestane in the absence and presence of 100 µM or 300 µM (*R*)-α-lipoic acid. The addition of (*R*)-α-lipoic acid has no significant effect on the IC_50_ of exemestane. A volume of 100 µM (*R*)-α-lipoic acid did not inhibit cell proliferation in MCF-7 cells, whereas it slightly reduced proliferation (by 12%) in T47D cells. In contrast, 300 µM (*R*)-α-lipoic acid significantly reduced proliferation in both cell lines with a more pronounced effect in T47D, confirming the data from Figure 2.

Based on the cytotoxic effects of 300 µM (*R*)-α-lipoic acid, we tested increasing concentrations of (*R*)-α-lipoic acid in combination with 1 µM exemestane for five days to test for synergy using MTT assay. Figure 5 shows the effects of single compounds and of the combinations in MCF-7 and T47D cells. Except for the combination of 300 µM (*R*)-α-lipoic acid with 1 µM exemestane in MCF-7 cells (which was not more efficient than 300 µM (*R*)-α-lipoic acid alone), all other combinations were significantly more efficient than (*R*)-α-lipoic acid alone. The 1 µM of exemestane alone showed no (T47D) or only small (10.2% in MCF-7) inhibition of cell viability. We then tested for synergistic effects by comparing the sum of the effects of single treatments versus the combination treatments (Figure 5c) and by Chou–Talaly analysis (Figure 5d). Figure 5c shows that all combinations behave synergistically: the sum of the effects of single treatments (black bars) are significantly smaller than the effects of the combination treatments (white bars, Figure 5c), except for 300 µM (*R*)-α-lipoic acid in MCF-7 cells.

In addition, we performed a Chou–Talalay analysis [27,28] with the data from Figure 4 and Figure 5a,b. Results are presented in Figure 5d as combination index (CI) values. For concentrations of 1 µM exemestane and 500–1000 µM (*R*)-α-lipoic acid, CI values of <0.2 were found in MCF-7 and T47D cells, indicating strong synergy according to Chou–Talalay. In addition, 300 µM (*R*)-α-lipoic acid with 1 µM exemestane gave a CI of 0.55 in T47D but not MCF-7 cells, indicating synergy at even lower concentrations in T47D.

**Figure 6:**
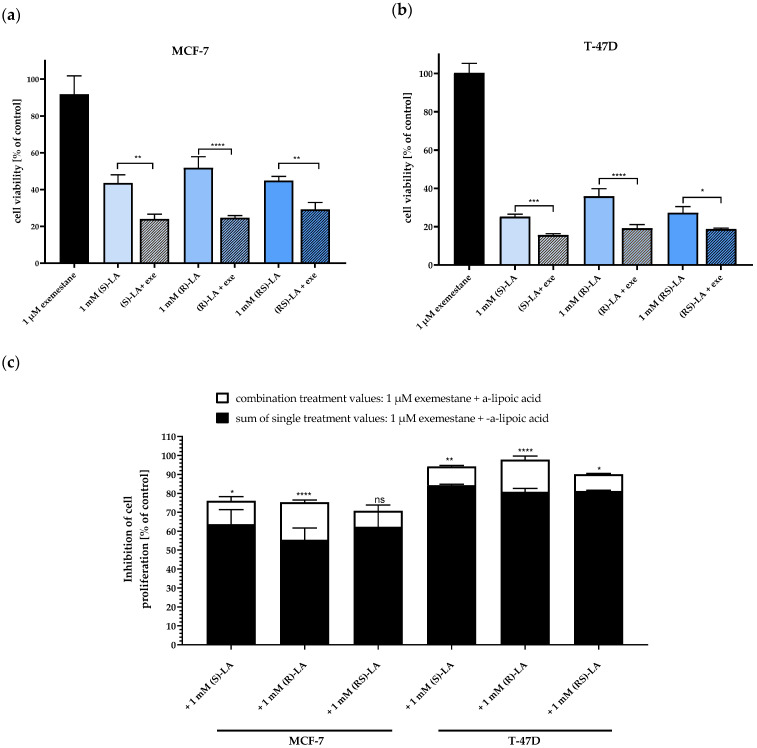
Combination treatment of 1 µM exemestane with 1 mM of (*R*)-, (*S*)-, and *rac*-α-lipoic acid. (**a**,**b**) show cell viability of MCF-7 (**a**) and T-47D (**b**) normalized to untreated control estimated via MTT assay after 120 h incubation. (**c**) Inhibition of cell viability based on data from (**a**,**b**): black bars represent the effects of the sum of single treatments, white bars illustrate the effect of the combination treatments. Data shown are mean ± SD of a representative experiment out of a series of 2 experiments, each carried out in triplicates. Statistical analysis was performed using a t-test. Levels of significance: * (*p* = 0.0108, 0.0268), ** (*p* = 0.0029), *** (*p* = 0.0002), **** (*p* < 0.0001). Exe = exemestane. (*R*)-LA, (*S*)-LA, (RS)-LA = (*R*)-, (*S*)-, and *rac*-α-lipoic acid. ns = not significant.

Next, we compared (*S*)-α-lipoic acid and racemic α-lipoic acid with (*R*)-α-lipoic acid in combination with 1 µM exemestane, respectively, to test if the S-enantiomer and the racemic mixture also show synergistic effects with exemestane as the R-enantiomer (Figure 5) does. Figure 6 presents cell viability data of the single compounds as well as the combinations. In accordance with the cytotoxicity data of the respective enantiomers of α-lipoic acid (Table 3), (*S*)-α-lipoic acid shows similar inhibition as (*R*)-α-lipoic acid in both cell lines. Further, as already shown for the R-enantiomer in Figure 5, also the S-enantiomer and the racemic mixture are significantly more effective in combination with exemestane than the respective α-lipoic acid enantiomer alone (Figure 6a,b). Furthermore, Figure 6c shows that in both cell lines, all combination treatments of exemestane and (*R*)-, (*S*)-, or *rac*-α-lipoic acid are synergistic except for the combination of *rac*-α-lipoic-acid plus 1 µM exemestane in MCF-7 cells, as can be derived from a significantly larger cytotoxic effect of the combination treatments (white bars) compared to the sum of the single treatments (black bars). Additionally, it can be concluded from these results that the synergistic effect of exemestane and α-lipoic acid in ER-positive breast cancer cells is not dependent on the stereochemistry of α-lipoic acid.

### 2.4. Effects of Exemestane and (R)-α-Lipoic Acid on Long-Term Proliferation

**Figure 7:**
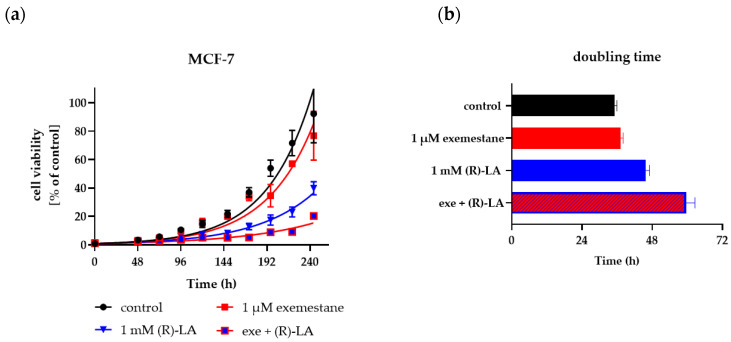
Cell proliferation of MCF-7 cells over 10 days. (**a**) Growth kinetics were analyzed of MCF-7 cells untreated (control) or treated with the respective compounds at the designated time points after staining with calcein AM and Hoechst 33342 using fluorescence microscopy. Data shown are percentage of cells stained by both calcein AM and Hoechst 33342 from 5 microscopic images per treatment and time point of one representative experiment out of a series of at least three experiments. (**b**) Doubling time was calculated for each treatment. Shown is mean and 95% confidence interval. Exe = exemestane. (*R*)-LA = (*R*)-α-lipoic acid.

**Figure 8:**
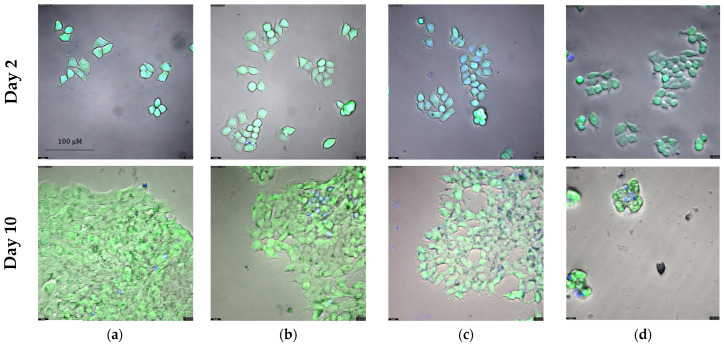
Fluorescence microscopic images of MCF-7 on day 2 (upper row) and day 10 (lower row). Shown are representative images from one experiment out of a series of at least 3 experiments. Cells are stained with calcein AM (green) and Hoechst 33342 (blue). Treatment conditions are (**a**) untreated control, (**b**) 1 µM exemestane, (**c**) 1 mM (*R*)-α-lipoic acid, (**d**) 1 µM exemestane and 1 mM (*R*)-α-lipoic acid. Magnification: 40×. Scale bar is shown in the upper left image and applies to all images.

In Section 2.3 we demonstrate that exemestane and α-lipoic acid are synergistic in ER-positive breast cancer cells. Here, we investigated the influence of single and combined treatment with exemestane and (*R*)-α-lipoic acid on the proliferation over 10 days. Cell growth was monitored after staining with calcein AM and Hoechst 33342, allowing for the quantification of the number of viable cells. Figure 7 and Figure 8 show results for MCF-7 cells. The exponential growth of MCF-7 is shown in Figure 7a and the corresponding doubling times in Figure 7b. All doubling times are significantly different from each other, although the difference between the untreated control and 1 µM exemestane is small. (*R*)-α-lipoic acid shows a strong reduction in proliferation and thus increase in doubling time which was further raised by the combination treatment of exemestane and (*R*)-α-lipoic acid. The inhibition of proliferation by the treatments is further illustrated in representative fluorescence microscopic images in Figure 8 taken on day 2 and day 10. On day 2, no evident differences can be observed between the various treatments, whereas on day 10, the number of cells was slightly reduced under exemestane treatment, clearly reduced with a kind of porous texture under (*R*)-α-lipoic acid treatment, and further reduced to only a few cells upon combination treatment (Figure 8, lower row).

**Figure 9:**
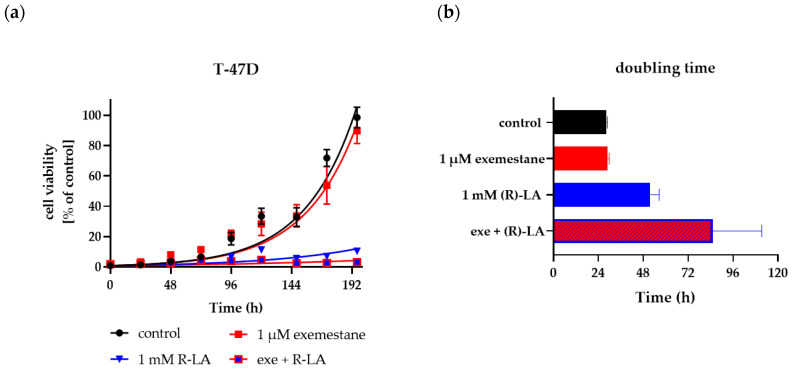
Cell proliferation of T47D cells over 10 days. (**a**) Growth kinetics were analyzed of T47D cells untreated (control) or treated with the respective compounds at the designated time points after staining with calcein AM and Hoechst 33342 via fluorescence microscopy. Data shown are the percentage of cells stained by both calcein AM and Hoechst 33342 from 5 microscopic images per treatment and time point of one representative experiment out of a series of at least three experiments. (**b**) Doubling time was calculated for each treatment. Shown is the mean and 95% confidence interval. Exe = exemestane. (*R*)-LA = (*R*)-α-lipoic acid.

**Figure 10:**
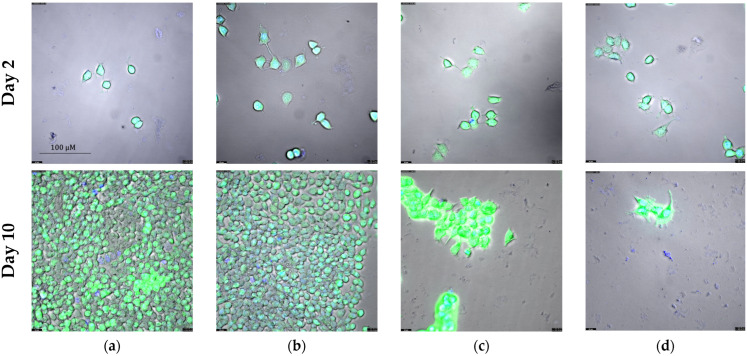
Fluorescence microscopic images of T47D on day 2 (upper row) and day 10 (lower row). Shown are representative images from one experiment out of a series of at least 3 experiments. Cells are stained with calcein AM (green) and Hoechst 33342 (blue). Treatment conditions are (**a**) untreated control, (**b**) 1 µM exemestane, (**c**) 1 mM (*R*)-α-lipoic acid, (**d**) 1 µM exemestane and 1 mM (*R*)-α-lipoic acid. Magnification: 40×. Scale bar is shown in the upper left image and applies to all images.

The same long-term incubation and proliferation experiment as with MCF-7 cells was also performed with T47D cells. Figure 9 and Figure 10 show the results for T47D cells. Exponential growth kinetics did not differ for untreated control and 1 µM exemestane-treated T47D cells (Figure 9a). Accordingly, the doubling time was not different between exemestane treatment and untreated condition (Figure 9b). This result is in accordance with data from Figure 5b where 1 µM exemestane for 120 h did not exert a cytotoxic effect. Treatment with 1 mM (*R*)-α-lipoic acid reduced proliferation and increased doubling time strongly. Combination treatment of exemestane and (*R*)-α-lipoic acid almost completely inhibited proliferation and increased the doubling time even further (Figure 9). Also, these data are in accordance with the results of Figure 5b. Figure 10 shows representative fluorescence images of live T47D cells (calcein AM and Hoechst 33342 staining), untreated or with single or combination treatment of exemestane and (*R*)-α-lipoic acid. Images were taken on day 2 (upper row) and day 10 (lower row). On day 2, no difference between untreated cells and any of the treatments was found. After 10 days, exemestane treatment alone gave a similar image as the untreated control. (*R*)-α-lipoic acid treatment alone led to a strong reduction in the cell number. Upon combination of (*R*)-α-lipoic acid with exemestane, only very few live cells were detected (Figure 10d, lower row). These results in T47D (Figure 10) as well as in MCF-7 (Figure 8) demonstrate again the strong effect of (*R*)-α-lipoic acid in reducing cell proliferation, particularly in combination with exemestane.

### 2.5. Caspase 3/7 Is Involved in Synergism between Exemestane and (R)-α-Lipoic Acid

**Figure 11:**
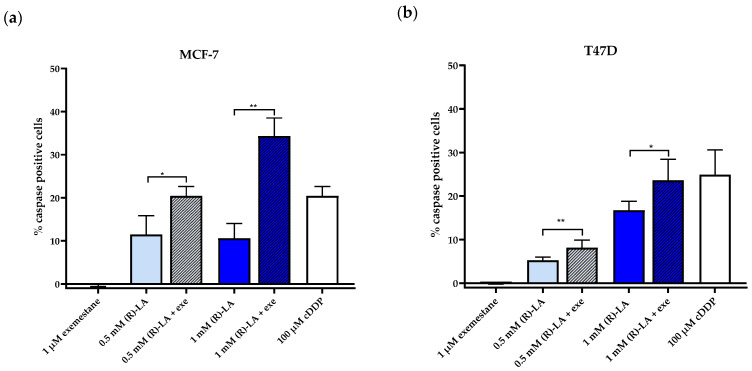
Caspase 3/7-activation by exemestane, (*R*)-α-lipoic acid, and their combination. MCF-7 (**a**) and T47D (**b**) cells were incubated with the given compounds for 24 h. Then, caspase 3/7-activation was monitored. A sample of 100 µM cisplatin for 24 h incubation served as positive control. Data shown are mean ± SD, *n* = 4. Statistical analysis was performed using t-test. Levels of significance: * (*p* ≤ 0.04); ** (*p* ≤ 0.01). Fluorescence images can be found in Figure S3a (MCF-7) and Figure S3b (T-47D). Exe = exemestane. (*R*)-LA = (*R*)-α-lipoic acid.

Next, we investigated if caspase 3/7 activation plays a role in the synergism between exemestane and (*R*)-α-lipoic acid. MCF-7 and T47D cells were treated for 24 h with exemestane or (*R*)-α-lipoic acid or the combination of both compounds (Figure 11). Longer incubation periods (48 h, 72 h) resulted in a decrease in caspase 3/7 activation. The 1 µM exemestane induced no caspase 3/7 activation. Combination treatment of 1 µM exemestane with 0.5 mM or 1 mM (*R*)-α-lipoic acid resulted in a significantly increased caspase activation compared to (*R*)-α-lipoic acid treatment alone in both cell lines (Figure 11), synonymous with a synergistic caspase 3/7 activation of the combination treatment. In MCF-7 cells, the combination treatment resulted in approximately 3-fold higher caspase activation than 1 mM (*R*)-α-lipoic acid alone and even exceeded caspase activation of 100 µM cisplatin serving as control. In T47D cells, the synergistic combination treatment reached the caspase activation level of 100 µM cisplatin (Figure 11). Fluorescent images showing caspase activation of untreated, single compound, and combination treatment together with cisplatin as control can be found in Supplemental Figure S3a,b.

Caspase activation by (*S*)-α-lipoic acid was slightly lower than (*R*)-α-lipoic acid which was in agreement with a somewhat similar cytotoxicity of (*S*)- and (*R*)-α-lipoic acid (Table 3).

### 2.6. Effects of Exemstane and (R)-α-Lipoic Acid Treatment on Expression of Selected Apoptosis-Related Proteins

**Figure 12:**
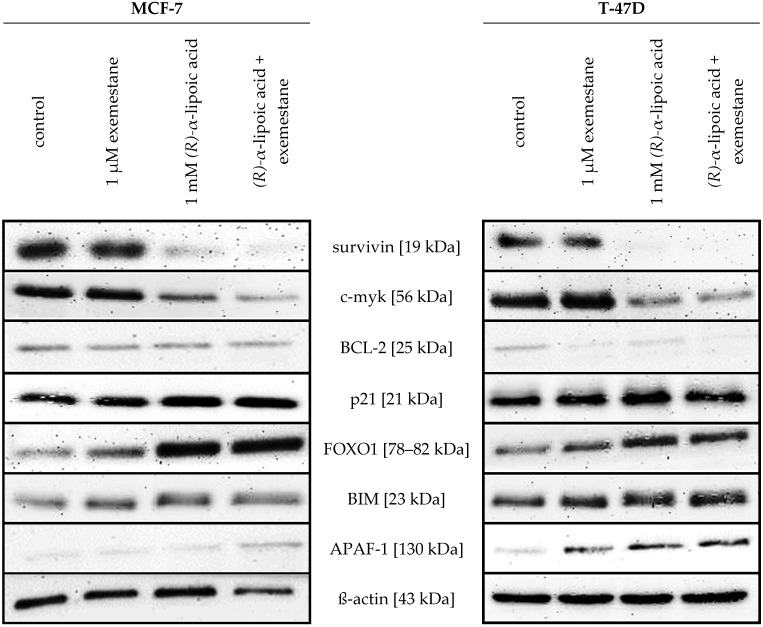
Effects of (*R*)-α-lipoic acid and exemestane on protein expression levels of apoptosis- and proliferation-related proteins in MCF-7 and T47D cells. Shown are representative immunoblots of survivin, c-myk, BCL-2, p21, FOXO1, BIM, APAF-1, and ß-actin (as loading control) in MCF-7 and T47D cells. Cells were treated for 48 h with the given compounds and concentrations. Uncropped and labeled immunoblots are presented in Figure S2.

**Figure 13:**
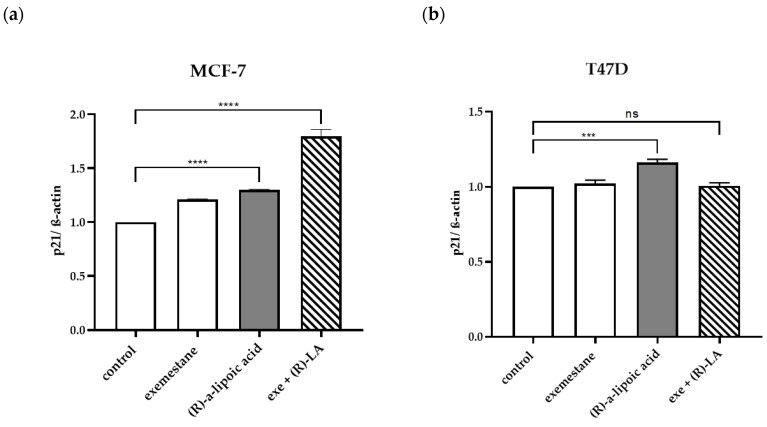
Densitometric analysis of the protein bands of p21 and ß-actin in MCF-7 (**a**) and T47D cells (**b**). Cells were treated 48 h with 1 µM exemestane, 1 mM (*R*)-α-lipoic acid, or the combination of both. Analysis was performed using ImageJ software version 1.54g/Java 1.8.0_345 (NIH, Bethesda, MD, USA). Data are mean ± SD, *n* = 3. All values have been normalized to MCF-7 or T-47D control. Statistical analysis was performed using a t-test. Levels of significance: *** (*p* = 0.0002); **** (*p* ≤ 0.0001). Exe = exemestane. (*R*)-LA = (*R*)-α-lipoic acid. ns = not significant.

Since α-lipoic acid moderately inhibits HDACs (see Table 1) and HDAC inhibition in cancer cells has been linked to changes in gene expression, we investigated the protein expression of apoptosis- and cell survival-related proteins upon 48 h treatment with the physiological R-enantiomer of α-lipoic acid, exemestane, and their combination in MCF-7 and T47D cells. Results are presented in Figure 12. Interestingly, the survival- and proliferation-promoting proteins survivin and c-myk were both strongly downregulated by (*R*)-α-lipoic acid and the combination of (*R*)-α-lipoic acid and exemestane, but not affected by exemestane alone in both cell lines. Additionally, anti-apoptotic BCL-2 was also downregulated. Cell cycle-inhibiting p21 was moderately upregulated by (*R*)-α-lipoic acid in both cell lines, which was additionally shown by densitometric analysis of the protein bands of p21 normalized to β-actin expression (Figure 13). Interestingly, the expression of p21 is even enhanced upon combination treatment of exemestane and (*R*)-α-lipoic acid in MCF-7 cells, but not in T47D. Pro-apoptotic proteins APAF-1 and BIM were upregulated, but particularly the apoptosis-inducing transcription factor FOXO1 was strongly upregulated upon (*R*)-α-lipoic acid treatment.

### 2.7. Analysis of the Mitochondrial Potential upon Treatment with (R)-α-Lipoic Acid

**Figure 14:**
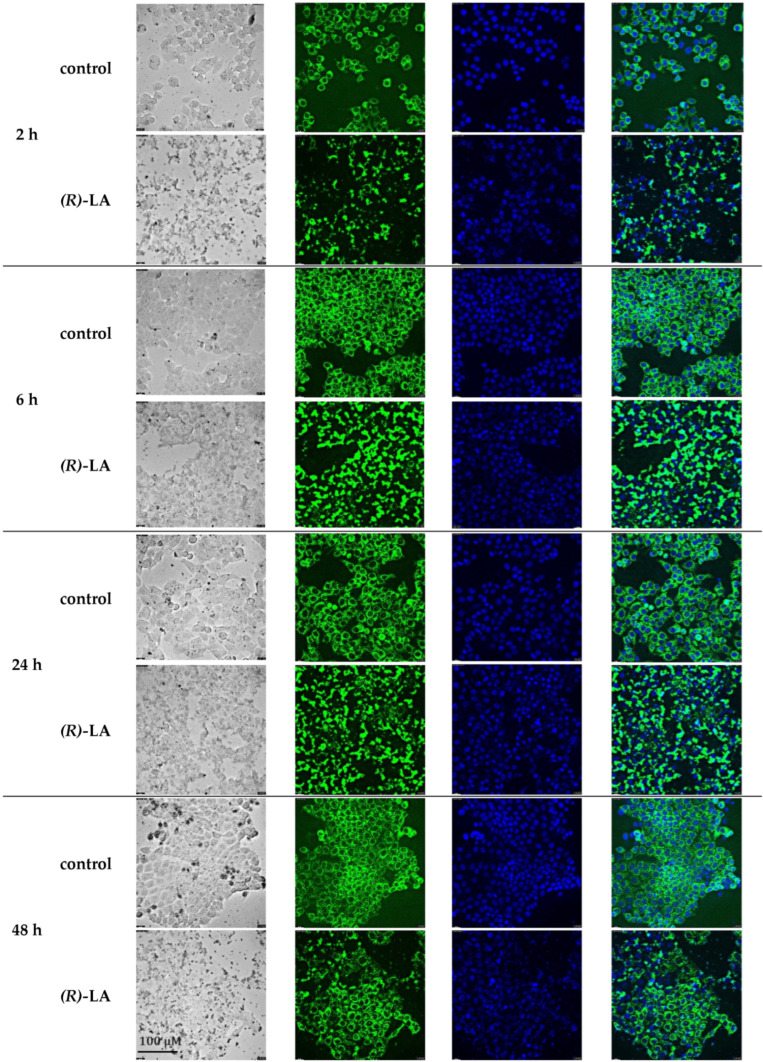
Kinetics of mitochondrial potential in MCF-7 cells upon treatment with (*R*)-α-lipoic acid. Displayed are representative fluorescence images (out of two independent experiments) of untreated control and 1 mM (*R*)-α-lipoic acid-treated MCF-7 cells after 2 h, 6 h, 24 h, and 48 h. Positive control with 20 µM CCCP is displayed in the Supplement Figure S4. First column shows brightfield microscope image of cells. Second column shows mitochondrial potential analyzed by TMRE (green) and third column, nuclei stained by Hoechst 33342 (blue). Fourth column: merged mitochondrial potential and nuclei. Magnification: 40×. Scale bar is shown in lower left image and applies to all images. (*R*)-LA = (*R*)-α-lipoic acid.

(*R*)-α-lipoic acid plays a major role in mitochondrial enzymes contributing to energy metabolism. Here, we investigated the role of (*R*)-α-lipoic acid on the function and structure of mitochondria. We used 1 mM (*R*)-α-lipoic acid, a concentration previously used to induce caspase activation (Figure 11), to inhibit cellular proliferation (Figure 7 and Figure 9), increase expression of pro-apoptotic proteins, and suppress anti-apoptotic proteins (Figure 12). Figure 14 shows representative brightfield and fluorescence microscopic images of MCF-7 cells untreated or treated with 1 mM (*R*)-α-lipoic acid after 2 h, 6 h, 24 h, and 48 h of incubation. Cells were stained with tetramethyl rhodamine ethylester (TMRE), allowing us to analyze the mitochondrial potential (green fluorescence), and nuclei were stained with Hoechst 33342 (blue). Untreated controls show functional and undamaged mitochondria (green) surrounding the blue-colored nuclei over the complete incubation time. Brightfield images also show undamaged cells with clear cellular boundaries. In contrast, cells treated with (*R*)-α-lipoic acid showed already after 2 h phenotypic aberrations in brightfield images and mitochondria staining. Brightfield images show scattered cells with some debris. Mitochondria give up their individual structure and show confluent structures around the nuclei while maintaining the intensity of the TMRE staining. The underlying mechanism is most likely a fusion of mitochondrial membranes of neighboring mitochondria for which GTPases are predominantly responsible. Certain membrane proteins such as mitofusin-2 cause the division protein Drp1 to move from the cytosol to the double membrane and increase the permeability of both the inner and outer membrane. The dynamin-like protein OPA1 then ensures that the inner mitochondrial membranes and cristae fuse. The membrane proteins Mfn1 and Mfn2 are responsible for the fusion of the outer membranes. This mitochondrial fusion culminates 6–24 h after treatment with (*R*)-α-lipoic acid. Presumably, the high concentration of 1 mM (*R*)-α-lipoic acid triggers pronounced stress on the cancer cells so that neighboring mitochondria fuse in order to respond to the environmental stress and trigger post-translational modifications. This is in accordance with the literature reporting that up to a certain level of damage, a network of mitochondria can provide compensation [33]. Since caspase 3/7 was shown to be activated after 24 h (Figure 11), it can be assumed that the integrity of the fused mitochondria seen up to 24 h will become disrupted. This can exactly be observed after 48 h. Mitochondrial fission occurs, partially with decreased TMRE staining, a clearly reduced number of cells (less blue-stained nuclei), and increasing accumulation of debris in the brightfield image (48 h). The division of previously fused mitochondria is also due to dynamics of mitochondrial proteins and proteins localized in the outer membrane (e.g., Drp1) binding to their membrane receptors Mff, Fis1, and MiD49/MiD 51 [34]. After anchoring to the outer membrane, Drp1 ensures the fragmentation of the membranes with the help of GTP hydrolysis. In contrast, untreated cells show a high confluence of regular, well-confined cells with intact mitochondrial TMRE staining (48 h). Taken together, analysis of mitochondrial potential confirms that treatment with 1 mM (*R*)-α-lipoic acid leads to mitochondrial stress as observed in mitochondrial fusion up to 24 h followed by mitochondrial fission and inhibition of proliferation (Figure 14), which is in accordance with our results from caspase activation (Figure 11) and inhibition of cell proliferation (Figure 7 and Figure 9).

### 2.8. Docking of (R)- and (S)-α-Lipoic Acid into a Molecular Model of HDAC6

Molecular docking studies were conducted to evaluate the binding affinities of the enantiomers of α-lipoic acid and dihydro-lipoic acid upon binding to HDAC6 from a structural level.

**Figure 15:**
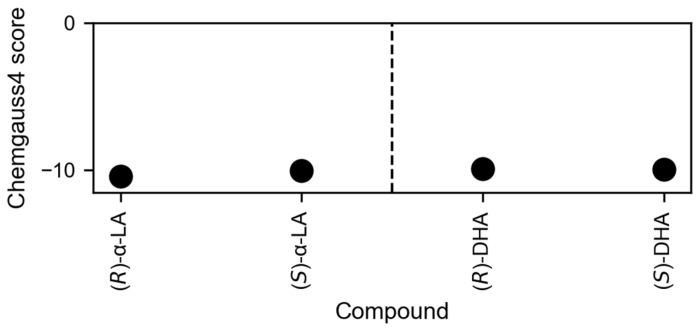
Docking scores of (*R*)-α-lipoic acid ((*R*)-α-LA), (*S*)-α-lipoic acid ((*S*)-α-LA), (*R*)-dihydro-lipoic acid ((*R*)-DHA), and (*S*)-dihydro-lipoic acid ((*S*)-DHA). The HYBRID Chemgauss4 scores for the fully deprotonated molecules are shown. The differences between R- and S-forms are marginal, for either the oxidized or reduced forms.

**Figure 16:**
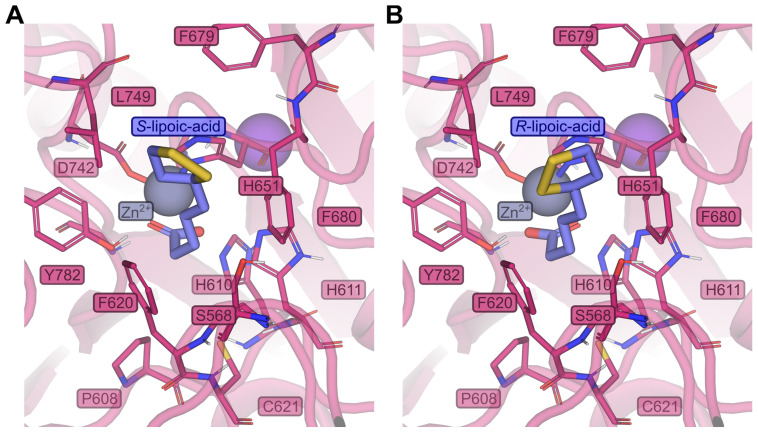
Predicted binding poses of (**A**) (*R*)- and (**B**) (*S*)-α-lipoic acid. The best docking pose of each structure (blue) out of 100 docking poses in human HDAC 6 (dark pink) is shown. Residues in close vicinity to the bound α-lipoic acid are shown as sticks. The grey sphere indicates the zinc ion, the magenta sphere, the potassium ion.

The HYBRID Chemgauss4 score, a scoring function that evaluates ligand poses in their binding site by using Gaussian smoothed potentials, is −10.4 for (*R*)-α-lipoic acid, −10.0 for (*S*)-α-lipoic acid, −9.9 for (*R*)-α-dihydro-lipoic acid, and −10.0 for (*S*)-α-dihydro-lipoic acid, respectively (Figure 15). Hence, the differences between enantiomer pairs are marginal, in line with HDAC inhibitory activities that differ by at most a factor of 1.5 and in the cellular context do not differ at all. Still, the interpretation of these results requires caution, since molecular docking and docking scores may provide valuable insights but their informative value for a quantitative comparison of the binding affinities of enantiomer pairs might be limited [35]. In addition to assessing the docking scores, the binding poses were visually inspected (Figure 16) for different interactions with the amino acids in the binding pocket. Note that according to the HYBRID docking approach, docking poses are similar to the dihydro-lipoic acid template in PDB ID 8TQ0. Still, lipoic acid docked to the zinc ion with its carboxylate moiety due to the low coordination potential of the dithiolane ring, which agrees with DTA-TGA-analysis, DFT calculations, and other docking studies [36]. The binding poses reveal in general no clear preference for one enantiomer, which supports the above results. Although the dithiolan rings in α-lipoic acid enantiomers or sulfanyl-ethyl moieties in dihydro-lipoic acid enantiomers point in opposite directions in the binding pocket (Figure 16), in neither case does the interaction profile of the sulfur atoms or thiol groups support a preference for one enantiomer over the other. By examination of the shape of the binding pocket, a slight steric hindrance of (*S*)-α-lipoic acid can be recognized (Figure S5B), which is also reflected in the clash score term of the scoring function ((*S*)-α-lipoic acid has a higher penalty score of 0.41 compared to 0.36 for (*R*)-α-lipoic acid) but which might be overcome if the protein structure is allowed to relax. Solely the proximity to tyrosine 782 (Y782) could explain the marginally better score of (*R*)-α-lipoic acid. (*S*)-α-lipoic acid is 0.6 Å closer to Y782 than (*R*)-α-lipoic acid, which may lead to a stronger disfavorable desolvation. This is reflected in the ProDesolv term of the scoring function, which is 0.76 for (*S*)-α-lipoic acid and 0.60 for (*R*)-α-lipoic acid. To conclude, the binding affinities and modes of the enantiomer pairs are very similar. This remains true when comparing the docking results for all protonation states and enantiomers for α-lipoic acid and dihydro-lipoic acid (Figure S6).

## 3. Discussion

Endocrine therapy is among the most effective treatments for ER-positive breast cancer. However, resistance emerges with cancer progression [6] and affects around 50% of all patients [37]. A newer approach to address resistance and improve therapeutic outcome is the combination of endocrine therapy with CDK4/6 inhibitors, particularly in advanced ER-positive breast cancer [38]. In addition to altered kinase signaling, aberrant epigenetic changes, in particular increased HDAC expression, as recently added as a new hallmark of cancer [37], contribute to endocrine therapy resistance [39,40]. Preclinical and clinical studies using HDACi with exemestane have shown partially conflicting results: a phase III trial in advanced ER-positive breast cancer with exemestane and entinostat could not confirm improved survival compared to exemestane alone [16], whereas in a Chinese phase III study the same combination significantly improved progression-free survival [17], which was also found for the combination of exemestane and tucidinostat [18]. However, these studies have revealed grade ≥ 3 adverse effects in the entinostat group [17], leaving the need for less toxic epigenetic modifiers. Here, we came across publications reporting dietary compounds as HDACi, particularly α-lipoic acid [19,20], an approved drug for the treatment of diabetic polyneuropathy, a food supplement, and with reported pleiotropic anticancer effects [23,24,25,26]. α-lipoic acid is rather non-toxic with a very low side-effect profile, particularly compared to other HDAC inhibitors [41]. First, we tested the enantiomers and the racemate of α-lipoic acid as well as the racemate of dihydro-lipoic acid at recombinant HDAC enzymes representative for the various HDAC classes. (*S*)-α-lipoic acid turned out to be an HDACi with comparable potency to (*R*)-α-lipoic acid. (*S*)-α-lipoic acid obtained an IC50 of 21.3 µM at HDAC6 (Table 1). Shortly after obtaining these results, Lechner et al. and later Watson et al. published that α-lipoic acid targets HDACs and that the most potent HDACi in the α-lipoic acid family is the reduced form of racemic or (*R*)-α-lipoic acid, i.e., dihydro-lipoic acid [21,22]. In addition, they reported that (*S*)-α-lipoic acid has no HDAC inhibitory activity up to 500 µM. Watson et al. described a crystal structure of HDAC6 with dihydro-lipoic acid [22]. Table 2 summarizes their and our IC50 values. The obvious contrasts are as follows: (*S*)-α-lipoic acid has similar potency to (*R*)-α-lipoic acid or *rac*-α-lipoic acid, but is inactive in the study of Lechner et al. up to 500 µM (max. concentration tested); *rac*-dihydro-lipoic acid is more active than *rac*-α-lipoic acid in Lechner et al.’s study: at least 5-fold at HDAC8 and around 100-fold at HDAC6. In our study, *rac*-dihydro-lipoic acid is also more active than the α-lipoic acid, but only slightly more active: 2.1-fold at HDAC2, 2.2-fold at HDAC6, and 2.9-fold at HDAC8. These differences prompted us to secure the identity and purity of our used α-lipoic acid derivatives. We obtained certificates of analysis from the manufacturers confirming enantiomeric purity (enantiomeric excess = 96.1% for (*S*)-α-lipoic acid and 97.78% for (*R*)-α-lipoic acid) of the compounds. Further, we performed high-resolution mass spectrometry and 1H-NMR analysis to confirm the molecular weight and purity of the compounds (see Supplemental Figure S1b). These analytical results confirmed the molecular weight, the identity, and the purity of our α-lipoic acid derivatives used in this study. Reference HDACi tubastatin A for HDAC6, panobinostat for HDAC4 and HDAC8, and vorinostat for HDAC2 confirmed the validity of our HDAC assays. In addition, we performed a docking of the α-lipoic acid and *rac*-dihydro-lipoic acid enantiomers into a molecular model of HDAC6. The binding affinities and modes of the enantiomer pairs are very similar which remains even true when comparing the docking results for all protonation states and conformers (see Figure 15, Supplemental Figure S6). Our HDAC enzyme results are thus backed by the outcomes of our molecular modeling study. At this point, we are unable to explain the contrasting results regarding (*S*)-α-lipoic acid and *rac*-dihydro-lipoic acid from Lechner et al.’s and our studies. However, since the major part of our cellular study uses (*R*)-α-lipoic acid in combination treatments with exemestane, and since the majority of α-lipoic acid is intracellularly reduced to *rac*-dihydro-lipoic acid, the discrepancy in HDAC inhibition between Lechner et al. and our data (Table 4) is of minor importance for the main findings of our study. Further, we could demonstrate that (*R*)-α-lipoic acid acts as a HDACi in cells by the treatment of MCF-7 and T47D cells with (*R*)-α-lipoic acid and monitoring an increase in acetylated histone and acetylated tubulin (Figure 1).

**Figure 17:**
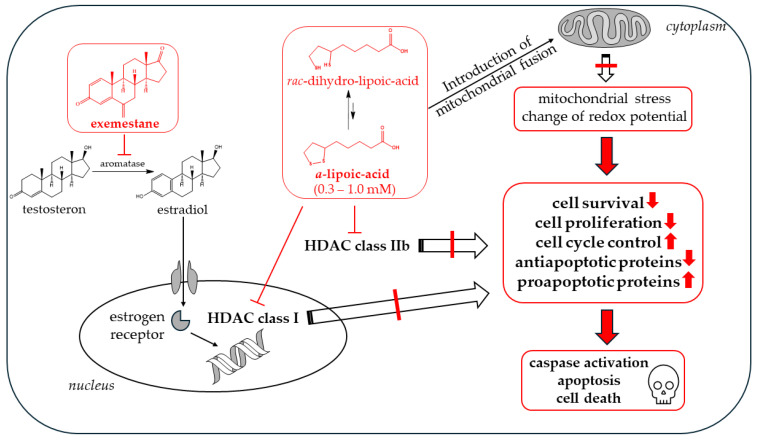
Mechanistic effects of the combination treatment of exemestane and α-lipoic acid in ER-positive breast cancer cells. Illustrated are the structures of exemestane and α-lipoic acid including its intracellular reduction to dihydro-lipoic acid with their effects on mitochondria and cell proliferation/survival, eventually leading to synergistic activation of apoptosis and cell death.

Our main finding is that α-lipoic acid is synergistic with exemestane. This was demonstrated in MTT cytotoxicity assays analyzed according to Chou–Talalay [27] (Figure 5), in fluorescence image analysis (Figure 7 and Figure 9), and in caspase activation assays in the ER-positive breast cancer cell lines MCF-7 and T47D (Figure 11). Exemestane and α-lipoic acid show a time-dependent, moderate to low cytotoxicity. Increasing the incubation time reduces IC_50_ values (72 h Table 2, 120 h Table 3). As expected, the lowest activity of exemestane was observed in MDA-MB-231 cells, a triple-negative breast cancer cell line serving as control for the ER-positive cells. α-lipoic acid and exemestane are synergistic as can be seen from Figure 5c where the sum of the cytotoxic effects of exemestane only and of α-lipoic acid only (black bars) is significantly smaller than the cytotoxic effect of the combination treatment. Fluorescence images demonstrate the additional effect of α-lipoic acid on exemestane, particularly after longer (10 day) incubation in MCF-7 (Figure 8) and T47D cells (Figure 10). Combining exemestane and α-lipoic acid (Figure 8d and Figure 10d) leaves only very few cells alive after 10 days treatment, whereas with each mono-treatment, a substantial number of cells survived (Figure 8b,c and Figure 10b,c). Inspection of the growth kinetics over 10 days revealed significantly different doubling times for all treatment conditions (untreated, exemestane-only, α-lipoic acid-only, combination of exemestane plus α-lipoic acid) in MCF-7 (Figure 7) and in T47D (Figure 9) except that the doubling time for control and exemestane was not different in T47D. These results confirm the synergy analysis in Figure 5 and emphasize the benefit of a combination of exemestane and α-lipoic acid. The combination of exemestane and α-lipoic acid induces caspase activation (Figure 11). Whereas 1 µM exemestane alone induces no caspase activation, and 0.5 mM or 1 mM α-lipoic acid induce some (5–15%) caspase activation, the combination of both compounds was synergistic in MCF-7 and T47D. Of note, in MCF-7 cells, the combination treatment increased caspase activation by 3-fold compared to (*R*)-α-lipoic acid alone, which is an even larger effect than previously observed by us with approved HDACi (panobinostat, entinostat) combined with cisplatin in ovarian cancer cells [11]. Mechanistically, α-lipoic acid leads to a loss or massive reduction in expression of the pro-survival proteins survivin, c-myk, and BCL-2. On the other hand, cell cycle-arresting p21 and pro-apoptotic FOXO1, BIM, and APAF-1 were increasingly expressed upon treatment with α-lipoic acid (Figure 12). By suppressing pro-survival and upregulating pro-apoptotic genes, α-lipoic acid may lead to increased caspase activation, eventually leading to decreased cell viability. These data may explain reduced proliferation in the long-term (10 day) proliferation experiment of α-lipoic acid-treated cells, particularly the increased doubling time (Figure 7 and Figure 9). Figure 17 summarizes the effects of exemestane and α-lipoic acid on their respective targets and resulting cellular effects. We and others have observed similar changes in protein expression in previous studies with synthetic HDACi [42,43]. In addition, α-lipoic acid induces mitochondrial changes (Figure 14). Already after 2 h incubation with 1 mM (*R*)-α-lipoic acid, mitochondrial fusion occurs, lasting until 24 h of incubation, most likely indicating mitochondrial stress induction [33]. After 48 h of incubation, mitochondrial fission, a clearly lower number of cells compared to untreated control, and reduced cell–cell contact can be recognized, indicating inhibition of proliferation (Figure 14 and Figure 17). In addition, we demonstrated caspase activation under the same conditions (Figure 11), supporting that mitochondrial stress leads to caspase activation and eventually inhibition of proliferation. Together, these data demonstrate that α-lipoic acid acts at least in part via HDAC inhibition (reduction in pro-survival genes, increase in anti-apoptotic genes, Figure 12) and furthermore via induction of mitochondrial stress (Figure 14), eventually acting synergistic (Figure 5) with exemestane (Figure 17). Additionally, α-lipoic acid may have further (pleiotropic) anticancer effects as reported in the literature [23,24,25,26].

One drawback in the clinical application of α-lipoic acid may be that high plasma concentrations of α-lipoic acid will be needed for a synergistic effect with exemestane in ER-positive breast cancer due to the low cytotoxic potency of α-lipoic acid. In our preclinical study, we used α-lipoic acid concentrations in the range of 300 to 1000 µM. Synergism was already observed with doses as low as 300 µM (Figure 5). Peak plasma concentrations obtained after a single oral dose of 300 mg (*R*)-α-lipoic acid were around 7 mg/L, corresponding to 34 µM [44]. Intravenous application of 600 mg α-lipoic acid gave peak plasma concentrations of around 100 µM [45]. Since α-lipoic acid has very low toxicity with a very low side-effect profile, particularly compared to other HDAC inhibitors [41], higher doses of α-lipoic acid may be applicable achieving higher blood levels. Alternatively, dermal (local) application, e.g., in non-metastasized breast cancer, might be a strategy to achieve locally high doses of α-lipoic acid.

In summary, the combination of exemestane and α-lipoic acid is synergistic under various incubation conditions (24 h to 10 d). α-lipoic acid induces mitochondrial fusion, increases the expression of apoptosis-related proteins p21, APAF-1, BIM, FOXO1, and decreases the anti-apoptotic proteins such as survivin, BCL-2, and c-myc, followed by caspase activation.

## 4. Materials and Methods

### 4.1. Reagents

Micronized exemestane was acquired from Farmabios S.p.A. (Gropello Cairoli, Italy) and dissolved in dimethyl sulfoxide in a concentration of 10 mM (Batch: 21430M0). (*R*)-α-lipoic acid was purchased by MedChemExpress (Princeton, NJ, USA) (Cat. #HY-18733/CS-5076). The enantiomer (*S*)-α-lipoic acid (CAS 1077-27-6), the racemic mixture *rac*-α-lipoic acid (CAS 1077-28-7), and *ras*-dihydro-lipoic acid (CAS 462-20-4) were purchased by Biosynth (Biosynth s.r.o., Bratislava, Slovakia).

### 4.2. Cell Lines and Cell Culture

The hormone-sensitive adenocarcinoma breast cancer cell line MCF-7, which kindly was provided by Dr. R. Hartmann, University of Saarbrücken, Germany, and the triple negative breast cancer cell line MDA-MB-231 (ATCC, Manassas, VA, USA, ATCC order number: ATCC^®^ HTB-26™) were cultivated in Dubecco’s Modified Eagle Medium (PAN Biotech GmbH, Aidenbach, Germany). DMEM was supplemented with 10% fetal calve serum (PAN Biotech) and 120 IU/mL penicillin and 120 µg/mL streptomycin (PAN Biotech GmbH, Aidenbach, Germany). The hormone-sensitive invasive ductal carcinoma breast cancer cell line T47D (ECACC, Salisbury, Wiltshire, UK) was cultivated in Roswell Park Memorial Institute Medium (RPMI 1640, ECACC, Salisbury, Whiteshare, UK) with the same amount of FCS and penicillin and streptomycin.

All cell lines were maintained in a humidified atmosphere of air with 5% CO_2_ at 37 °C.

### 4.3. MTT Cell Viability Assay

MTT assays were performed as previously described [46]. Cell numbers plated differed depending on each incubation time: MDA-MB-231 5000 c/w, MCF-7 5000 c/w (72 h) and 3000 c/w (120 h), T-47D 10,000 c/w (72 h) and 5000 c/w /120 h). After incubation time, medium was replaced by MTT (solution of 5 mg/mL 3-(4,5-dimethylthiazol-2-yl)-2,5-diphenyl-tetrazoliumbromidin in phosphate buffered saline, Serva, Heidelberg, Germany). After 10 min (MCF-7), 20 min (MDA-MB-231), and 30 min (T47D) and dissolving in DMSO (VWR, Langenfeld, Germany), absorbance was measured at 544 nm and 690 nm in a FLUOstar microplate reader (BMG LabTech, Offenburg, Germany).

### 4.4. Life Dead Proliferation Assay via Leica Microscope

Cells were seeded on day one in 96-well plates. On day two medium was changed and cells were treated with 1 µM exemestane, 1 mM (*R*)-α-lipoic acid, as well as with combination treatments of exemestane with (*R*)-α-lipoic acid and vehicle control. Then, cells were stained with calcein AM (Millipore Corporation, Billerica, MA, USA) and Hoechst 33342 (Tocris Bioscience, Bristol, UK) for 30 min and fluorescence images monitored by Leica DMi8 Thunder Imager (Leica Microsystems CMS GmbH, Wetzlar, Germany). Five images per well were monitored randomly. Calcein AM- and Hoechst 33342-stained area of the images (i.e., live proliferating cells) were evaluated.

### 4.5. Enzyme HDAC Inhibition Assay

All human recombinant enzymes were purchased from Reaction Biology Corp. (Malvern, PA, USA). The HDAC activity assays HDAC2 (cat nr. KDA-21-277), HDAC4 (cat nr. KDA-21-279), HDAC6 (cat nr. KDA-21-213), and HDAC8 (cat nr. KDA-21-481) were performed in 96-well plates (Corning, Kaiserslautern, Germany). First, 10 µL of increasing concentrations of α-lipoic acid were used, then 20 ng of HDAC2/8, 17.5 ng of HDAC6, and 2 ng of HDAC4 per well/reaction were diluted in assay buffer (50 mM Tris-HCl, pH 8.0, 137 mM NaCl, 2.7 mM KCl, 1 mM MgCl_2_, and 1 mg/mL BSA) were added. After 5 min, 10 µL of 300 µM (HDAC2) or 150 µM (HDAC 6) Boc-Lys(Ac)-AMC (Bachem, Bubendorf, Switzerland) or 100 µM (HDAC4) or 60 µM (HDAC8) Boc-Lys(TFA)-AMC (Bachem, Bubendorf, Switzerland) were added to each reaction. After 90 min incubation, the reaction was stopped by adding 100 µL stop solution (16 mg/mL trypsin, 4 µM vorinostat for HDAC2, 4 µM panobinostat for HDAC 4/8, and 4 µM tubastatin A for HDAC 6 in 50 mM Tris-HCl, pH 8.0, and 100 mM NaCl). After another 15 min, fluorescence intensity was measured at excitation of 355 nm and emission of 460 nm in a NOVOstar microplate reader (BMG LabTech, Offenburg, Germany).

### 4.6. Whole-Cell HDAC Inhibition Assay

The cellular HDAC assay was performed based on an assay published by Heltweg and Jung and Ciossek et al. [28,47]. Breast cancer cell line T-47D (12,000 c/w) was seeded in 96-well tissue culture plates (Corning, Kaiserslautern, Germany) in a total volume of 90 µL of culture medium. After 24 h, cells were incubated 48 h with increasing concentrations of *a*-lipoic acid. The reaction was started by adding 10 µL of 3 mM Boc-Lys(Ac)-AMC (Bachem, Bubensdorf, Switzerland) and again incubated for 3 h under cell culture conditions. Then, 100 µL/well stop solution (25 mM Tris-HCl (pH 8), 137 mM NaCl, 2.7 mM KCl, 1 mM MgCl_2_, 1% NP40, 2.0 mg/mL Trypsin, 10 µM vorinostat) was added and again incubated for 3 h. Fluorescence intensity was measured at excitation of 320 nm and emission of 520 nm in a NOVOstar microplate reader (BMG LabTech, Offenburg, Germany)

### 4.7. Caspase 3/7 Activation Assay

To analyze the compound-induced activation of capases 3 and 7, the CellEvent caspase 3/7 green detection reagent (Thermo Scientific, Wesel, Germany) was used according to the manufacturer’s instructions. MCF-7 (5000 c/w) and T47D (6000 c/w) cells were seeded in 96-well plates and treated the next day with 1 µM exemestane, 1 mM (*R*)-a-lipoic acid, a combination of these two, or (*S*)-α-lipoic acid and 100 µM cisplatin. After 24 h incubation, medium was removed, and cells were stained with 50 µL of CellEvent caspase 3/7 green detection reagent (2 µM in PBS supplemented with 5% heat-inactivated FBS). Cells were incubated for 30 min at 37 °C in a humidified incubator before imaging by using the Thermo Fisher ArrayScan XTI high content screening (HCS) system with a 10× magnification (Thermo Scientific). Hoechst 33342 was added for nuclei staining.

### 4.8. Immunoblotting

Cells were treated 48 h with indicated concentrations of exemestane, (*R*)-α-lipoic acid, the combination of these two, or vehicle, followed by dissolving cell pellets with RIPA-buffer (50 mM Tris-HCl pH 8.0, 1% Triton X-100, 0.5% sodium deoxycholate, 0.1% SDS, 150 mM sodium chloride, 2 mM EDTA, supplemented with protease and phosphatase inhibitors (Pierce^TM^ protease and phosphatase inhibitor mini tablets, Thermo Scientific, Wesel, Germany)) and clarification by centrifugation. A quantity of 20 or 40 µg of total protein as well as, from PageRuler Prestained Protein Ladder, 10–180 kDA (Thermo Scientific, Wesel, Germany), were resolved by SDS-Page (12% polyacrylamide gel) and transferred to polyvinylidene fluoride membrane (Millipore Corporation, Billerica, MA, USA). Blots were incubated with primary antibodies against acetylated a-tubulin (Cat. No. sc-23950), a-tubulin (Cat. No. sc-8035) (Santa Cruz Biotechnology, Heidelberg, Germany), acetyl histone h3 (Cat. No. 39140) and histone H3 (Cat. No. NB100-1669), ß-actine (Cat. No. sc-47778), p21 (AF1047), c-myk (Cat. No. MAB3696), APAF1 (Cat. No. MAB868), BIM (Cat. No. NBP1-76936), FOXO1 (Cat. No. C29H4), BCL-2 (Cat. No. AF810), and survivin (Cat. No. AF886). After another one-hour incubation with the appropriate HRP-conjugated secondary antibody, proteins were visualized by Intas Imager (Intas, Göttingen, Germany) using luminol reagent (Santa Cruz Biotechnology, Heidelberg, Germany).

### 4.9. Mitochondrial Assay

The analysis of mitochondrial potential was carried out as previously described with minor modifications concerning fluorescence measurement [48]. MCF-7 cells (13,000 c/w) were seeded in 96-well plates and treated the next day with 1 mM (*R*)-α-lipoic acid. After an incubation time of 2 h, 6 h, 24 h, and 48 h, medium was removed, and cells were washed with PBS and then stained for 30 min at 37 °C with 50 µL staining reagent containing 500 nM tetramethyl rhodamine ethylester as perchlorate (TMRE, BIOTREND chemicals GmbH, Cologne) and Hoechst 33342 (5 µg/mL). A quantity of 20 µM CCCP (carbonyl cyanide-m-chlorophenylhydrazone, Sigma-Aldrich, München, Germany) served as control for mitochondrial potential breakdown. Fluorescence was measured by Leica DMi8 Thunder Imager (Leica Microsystems CMS GmbH, Wetzlar, Germany).

### 4.10. Data Analysis

All bar charts and concentration–effect curves were created with Prism (GraphPad Prism 8.0.2, San Diego, CA, USA). Depending on the experiment, either individual experiments from three triplicates each were analyzed, or three experiments performed in triplicates were pooled and the data fitted to the four-parameter logistic equation. For statistical analysis, an unpaired t-test was used. Synergistic effects in Section 2.3 were analyzed based on previous methods by Bandolik et al. [11].

### 4.11. Molecular Docking Studies

Protonation states for (*R*)-α-lipoic acid, (*S*)-α-lipoic acid, (*R*)-dihydro-lipoic acid, and (*S*)-dihydro-lipoic acid were enumerated using the pKaTyper module of QUACPAC 2.2.4.0 [QUACPAC 2.2.4.0. OpenEye, Cadence Molecular Sciences, Santa Fe, NM, USA [49]]. A conformer library was then generated for each compound and pK_a_ microstate using OMEGA 5.1.0.0 [OMEGA 5.1.0.0. OpenEye, Cadence Molecular Sciences, Santa Fe, NM, USA [49]]. Molecular docking was performed using HYBRID from the OEDocking 4.3.1.0 suite of programs [OEDocking 4.3.1.0. OpenEye, Cadence Molecular Sciences, Inc., Santa Fe, NM, USA [49]] and a crystal structure of the human HDAC6 catalytic domain 2 bound to trichostatin A (PDB ID: 5EDU [50]) as an initial structure. To create a reference structure suitable for HYBRID, the (*R*)-α-lipoic acid-bound *Danio rerio* HDAC6 structure (PDB ID: 8TQ0 [22]) was superimposed onto the initial structure. The coordinates of trichostatin A from the initial complex were then replaced with the coordinates of (*R*)-α-lipoic acid from the superimposed structure. For each structure, a maximum of 100 poses were output.

## 5. Conclusions

This study revealed that the aromatase inhibitor exemestane and the antioxidant and HDAC α-lipoic acid are synergistic in caspase activation and inhibiting cell proliferation of the ER-positive breast cancer cell lines MCF-7 and T-47D. α-lipoic acid acts at least in part by inducing mitochondrial fusion, and increasing the expression of pro-apoptotic and reducing the expression of pro-survival proteins. In conclusion, combining exemestane with α-lipoic acid is a promising novel treatment option for ER-positive breast cancer.

## Figures and Tables

**Table 1:** HDAC inhibitory activity of (*R*)-, (*S*)-, and *rac*-α-lipoic acid, and the reduced racemic form *rac*-dihydro-lipoic acid. Enzyme HDAC assays were performed with HDAC2, 4, 6, and 8. Cellular HDAC assay was performed with the class I/IIb selective HDAC substrate Boc-Lys-AC-AMC in T47D cells. Panobinostat, vorinostat, and tubastatin A served as references. Shown are IC_50_ values ± SEM [µM]. HDAC enzyme assays were performed three times, each carried out in duplicates. Cellular HDAC assays are based on one experiment, carried out in triplicates. ND = not determined.

	HDAC 2	HDAC 4	HDAC 6	HDAC 8	Cellular HDAC Assay
(*R*)-α-lipoic acid	208 ± 34.9	168 ± 19.3	14.2 ± 2.62	151 ± 33.4	969 ± 120
(*S*)-α-lipoic acid	299 ± 34.6	324 ± 39.9	21.3 ± 1.23	559 ± 71.0	906 ± 87.7
*rac*-α-lipoic acid	208 ± 4.2	262 ± 37.8	15.3 ± 4.60	464 ± 83.0	1186 ± 67.0
*rac*-dihydro-lipoic acid	98.1± 35.6	177 ± 17.5	6.48 ± 2.11	51.5 ± 10.5	ND
panobinostat		1.56 ± 0.24		0.73 ± 0.16	
vorinostat	0.12 ± 0.01				0.79 ± 0.07
tubastatin A			0.07 ± 0.01		

**Table 2:** IC_50_ values ± SEM [µM] of (*R*)-α-lipoic acid and exemestane in MCF-7, T47D, and MDA-MB-231, determined by a 72 h MTT assay. Presented are IC_50_ values ± SEM [µM]. Data shown are means of pooled data of at least 2 experiments, all carried out in triplicates.

	MCF-7	T-47D	MDA-MB-231
(*R*)-α-lipoic acid	1167 ± 28.9	1119 ± 67.8	873 ± 36.0
exemestane	29.1 ± 1.30	50.6 ± 2.04	89.4 ± 2.86

**Table 3:** IC_50_ ± SEM [µM] of exemestane and (*R*)-, (*S*)- and *rac*-α-lipoic acid in MCF-7, T47D, and MDA-MB-231 cell lines, determined by a 120 h MTT assay. Data shown are means of pooled data of at least 2 experiments, all carried out in triplicates.

	MCF-7	T-47D	MDA-MB-231
exemestane	16.1 ± 0.84	28.7 ± 0.75	53.3 ± 3.62
(*R*)-α-lipoic acid	439 ± 16.3	435 ± 28.6	225 ± 7.33
(*S*)-α-lipoic acid	429 ± 26.8	338 ± 17.4	243 ± 25.1
*rac*-α-lipoic acid	454 ± 15.2	373 ± 13.9	275 ± 22.5

**Table 4:** HDAC inhibitory activity of (*R*)-, (*S*)- and *rac*-α-lipoic acid and the reduced racemic form *rac*-dihydro-lipoic acid from our group versus Lechner et al. [21] at HDAC2, 6, and 8. Shown are IC_50_ values [µM]. Our data are taken from Table 1, which also contains reference HDACi controls. IC_50_ values from Lechner et al. were obtained by fitting their source data to a sigmoidal concentration–effect model. n.e. = no effect.

IC_50_ [µM]		(*R*)-α-Lipoic Acid	(*S*)-α-Lipoic Acid	*rac*-α-Lipoic Acid	*rac*-Dihydro-Lipoic Acid
**HDAC 2**	Pradel et al.	208	299	208	98.1
	Lechner et al.	~ 500	n.e. up to 500 µM	391	27.9
**HDAC 6**	Pradel et al.	14.2	21.3	15.3	6.48
	Lechner et al.	210	n.e. up to 500 µM	191	1.70
**HDAC 8**	Pradel et al.	151	559	464	51.5
	Lechner et al.	127	n.e. up to 500 µM	109	22.3

## Data Availability

Data is contained within the article and Supplementary Materials.

## Supplementary Materials

The supporting information can be downloaded at: https://www.mdpi.com/article/10.3390/ijms25158455/s1.
