# Supplementary material for: The Antioxidant and HDAC-Inhibitor α-Lipoic Acid Is Synergistic with Exemestane in Estrogen Receptor-Positive Breast Cancer Cells
Source: Int J Mol Sci. 2024 Aug 2;25(15):8455. doi: 10.3390/ijms25158455 (PMC11313180; doi:10.3390/ijms25158455)
Supplement: Supplementary file 1 [file ijms-25-08455-s001.zip › ijms-3083340-supplementary.pdf]

Supplemental Information

Mass Spectrum SmartFormula Report

Analysis Info

Analysis Name	D:\Data\Spektren 2024\KAS24HR000003.d	Acquisition Date	6/7/2024 12:23:19 PM
Method	tune_low_new.m	Operator	PT
Sample Name	Pradel S-LA (CH3OH)	Instrument	maXis 288882.20213
Comment			

Acquisition Parameter

Source Type	ESI	Ion Polarity	Positive	Set Nebulizer	0.3 Bar
Focus	Not active	Set Capillary	4000 V	Set Dry Heater	180 °C
Scan Begin	50 m/z	Set End Plate Offset	-500 V	Set Dry Gas	4.0 l/min
Scan End	1500 m/z	Set Collision Cell RF	600.0 Vpp	Set Divert Valve	Source

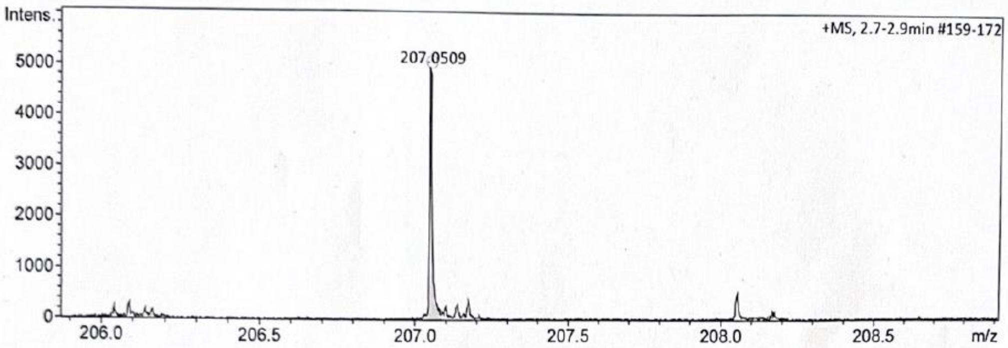

Meas. m/z	#	Ion Formula	m/z	err [ppm]	mSigma	# mSigma	Score	rdb	e⁻ Conf	N-Rule
207.0509	1	C8H15O2S2	207.0508	-0.7	n.a.	1	100.00	1.5	even	ok

Mass Spectrum SmartFormula Report

Analysis Info

Analysis Name	D:\Data\Spektren 2024\KAS24HR000001.d	Acquisition Date	5/31/2024 8:47:06 AM
Method	tune_low_new.m	Operator	PT
Sample Name	Pradel R-LA (CH3OH)	Instrument	maXis 288882.20213
Comment			

Acquisition Parameter

Source Type	ESI	Ion Polarity	Positive	Set Nebulizer	0.3 Bar
Focus	Not active	Set Capillary	4000 V	Set Dry Heater	180 °C
Scan Begin	50 m/z	Set End Plate Offset	-500 V	Set Dry Gas	4.0 l/min
Scan End	1500 m/z	Set Collision Cell RF	600.0 Vpp	Set Divert Valve	Source

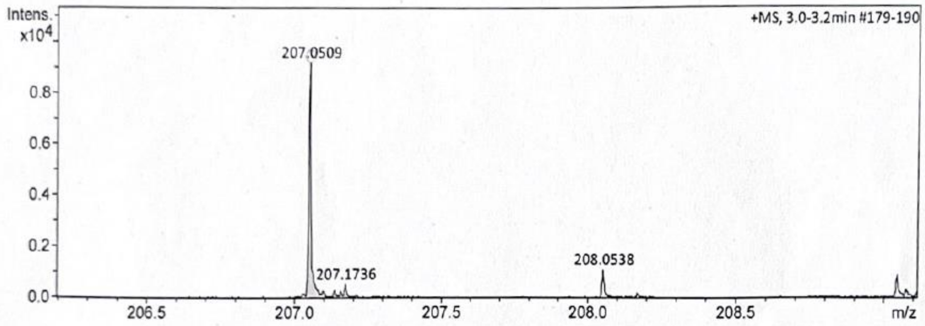

Meas. m/z	#	Ion Formula	m/z	err [ppm]	mSigma	# mSigma	Score	rdb	e⁻ Conf	N-Rule
207.0509	1	C8H15O2S2	207.0508	-0.3	10.9	1	100.00	1.5	even	ok

Mass Spectrum SmartFormula Report

Analysis Info

Analysis Name	D:\Data\Spektren 2024\KAS24HR000002.d	Acquisition Date	6/6/2024 3:39:42 PM
Method	tune_low_new.m	Operator	PT
Sample Name	Pradel RS-LA (CH3OH)	Instrument	maXis 288882.20213
Comment			

Acquisition Parameter

Source Type	ESI	Ion Polarity	Positive	Set Nebulizer	0.3 Bar
Focus	Not active	Set Capillary	4000 V	Set Dry Heater	180 °C
Scan Begin	50 m/z	Set End Plate Offset	-500 V	Set Dry Gas	4.0 l/min
Scan End	1500 m/z	Set Collision Cell RF	600.0 Vpp	Set Divert Valve	Source

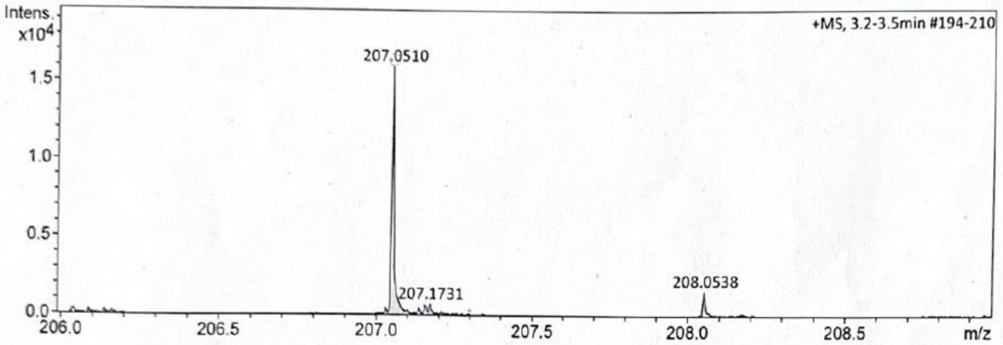

Meas. m/z	#	Ion Formula	m/z	err [ppm]	mSigma	# mSigma	Score	rdb	e⁻ Conf	N-Rule
207.0510	1	C8H15O2S2	207.0508	-1.1	5.4	1	100.00	1.5	even	ok

Mass Spectrum SmartFormula Report

Analysis Info

Analysis Name	D:\Data\Spektren 2024\KAS24HR000006.d	Acquisition Date	6/7/2024 12:44:07 PM
Method	neg_tune_low.m	Operator	PT
Sample Name	Pradel DH-RS-LA (CH3OH)	Instrument	maXis 288882.20213
Comment			

Acquisition Parameter

Source Type	ESI	Ion Polarity	Negative	Set Nebulizer	0.3 Bar
Focus	Not active	Set Capillary	2800 V	Set Dry Heater	200 °C
Scan Begin	50 m/z	Set End Plate Offset	-500 V	Set Dry Gas	4.0 l/min
Scan End	1650 m/z	Set Collision Cell RF	700.0 Vpp	Set Divert Valve	Waste

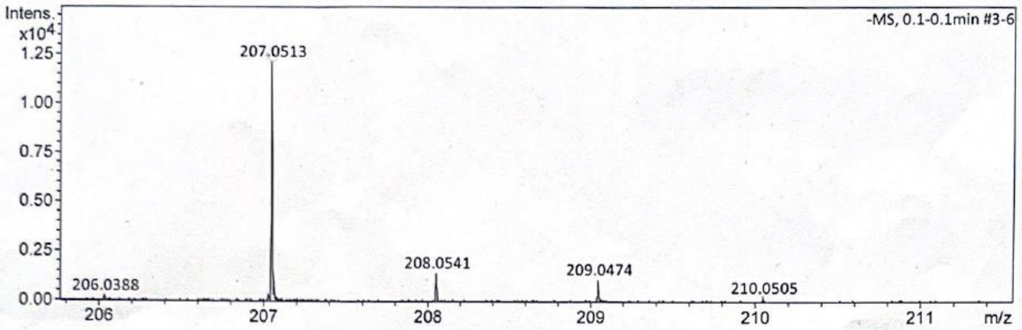

Meas. m/z	#	Ion Formula	m/z	err [ppm]	mSigma	# mSigma	Score	rdb	e⁻ Conf	N-Rule
207.0513	1	C8H15O2S2	207.0519	2.8	8.4	1	100.00	1.5	even	ok

Figure S1a. High-resolution mass spectrometry analysis of *(R)*-, *(S)*- and *rac*-α-lipoic acid as well as *rac*-dihydro-lipoic acid. Mass spectra were recorded in ESI mode and performed by the Center for Molecular and Structural Analytics of the Heinrich Heine University Duesseldorf.

a)

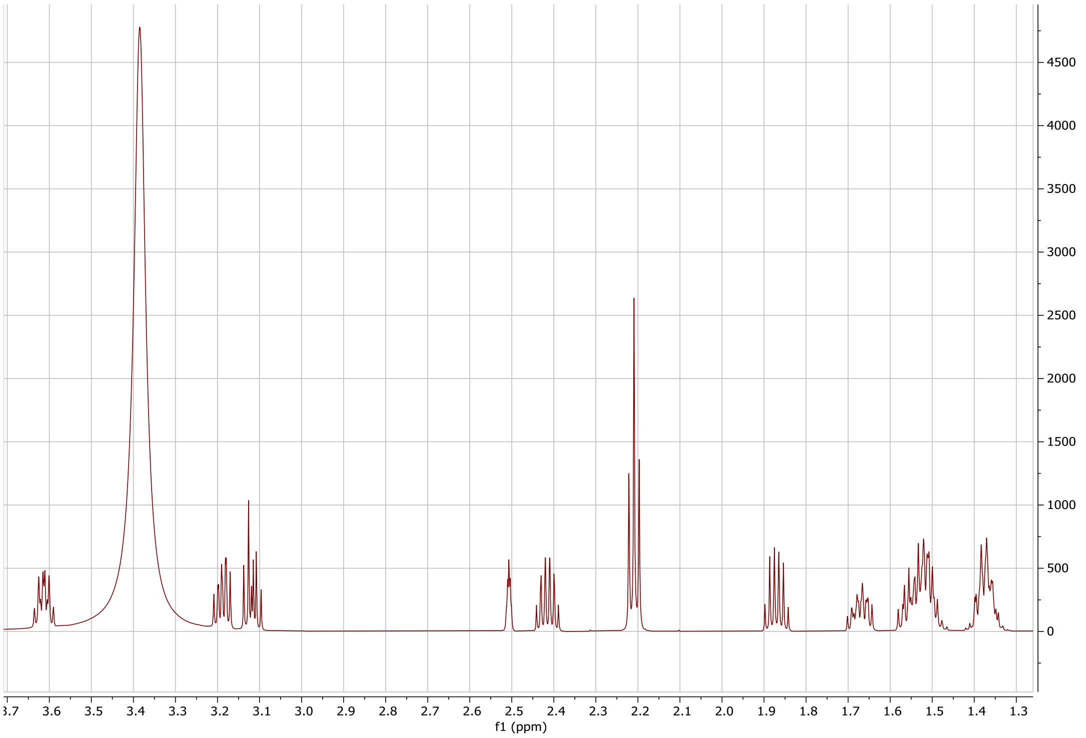

b)

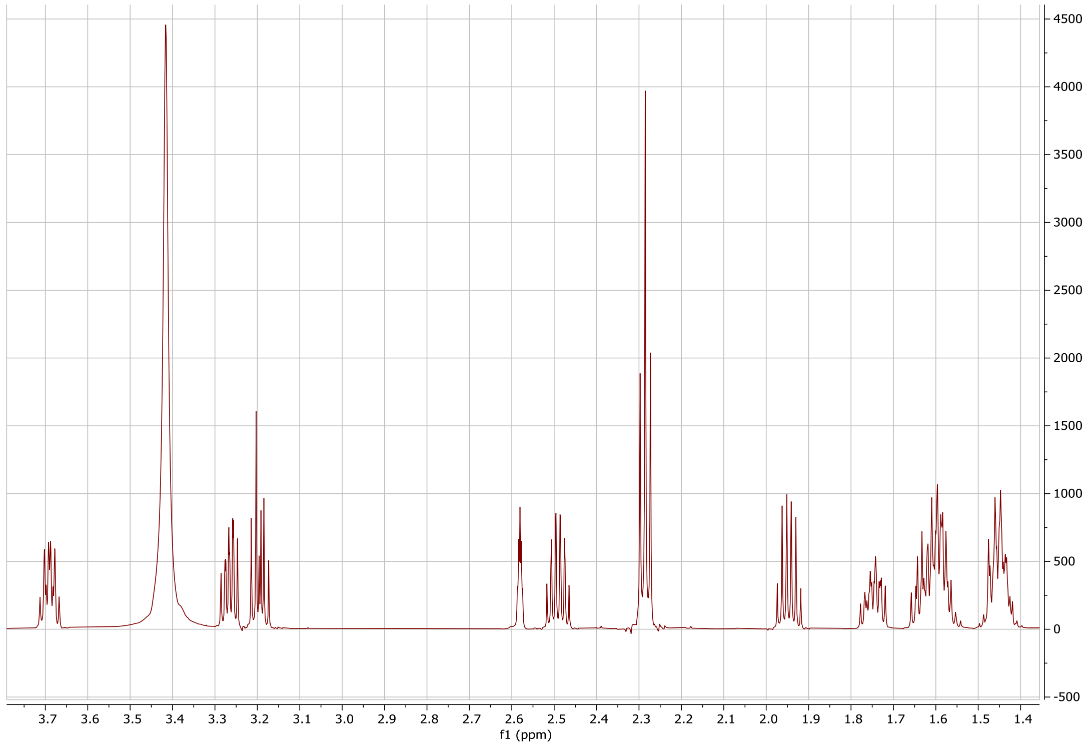

Figure S1b. ^{1}H-NMR spectra of the two enantiomers *(R)*-α-lipoic acid (a) and *(S)*-α-lipoic acid (b). Substances were solved in DMSO-d_6 and measured at 600 MHz. Peaks with a chemical shift of 2.58 and 3.42 ppm are due to solvent residues.

a)

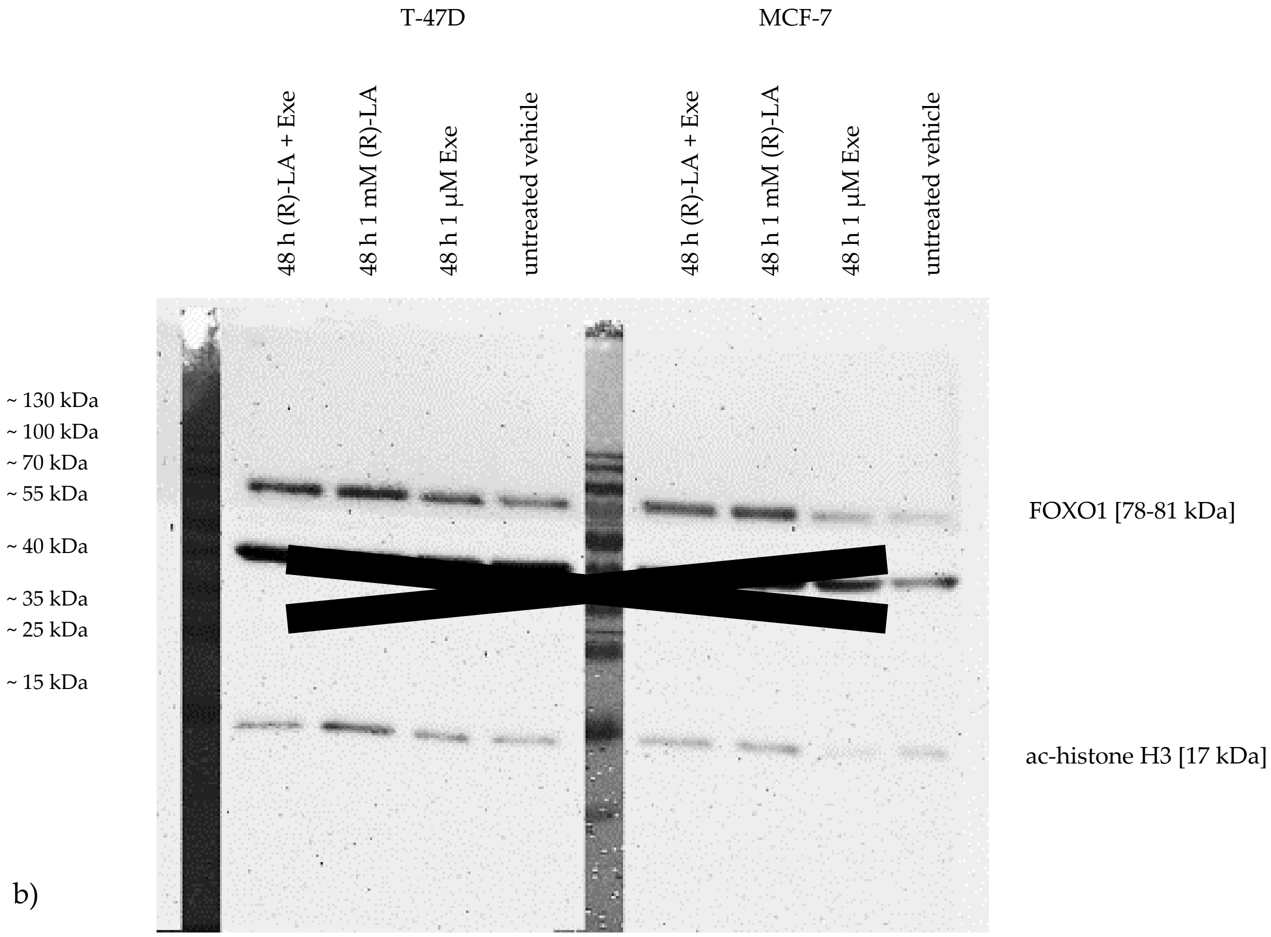
T-47D
MCF-7
48 h (R)-LA + Exe
48 h 1 mM (R)-LA
48 h 1 μM Exe
untreated vehicle
48 h (R)-LA + Exe
48 h 1 mM (R)-LA
48 h 1 μM Exe
untreated vehicle
~ 130 kDa
~ 100 kDa
~ 70 kDa
~ 55 kDa
~ 40 kDa
~ 35 kDa
~ 25 kDa
~ 15 kDa
FOXO1 [78-81 kDa]
ac-histone H3 [17 kDa]

b)

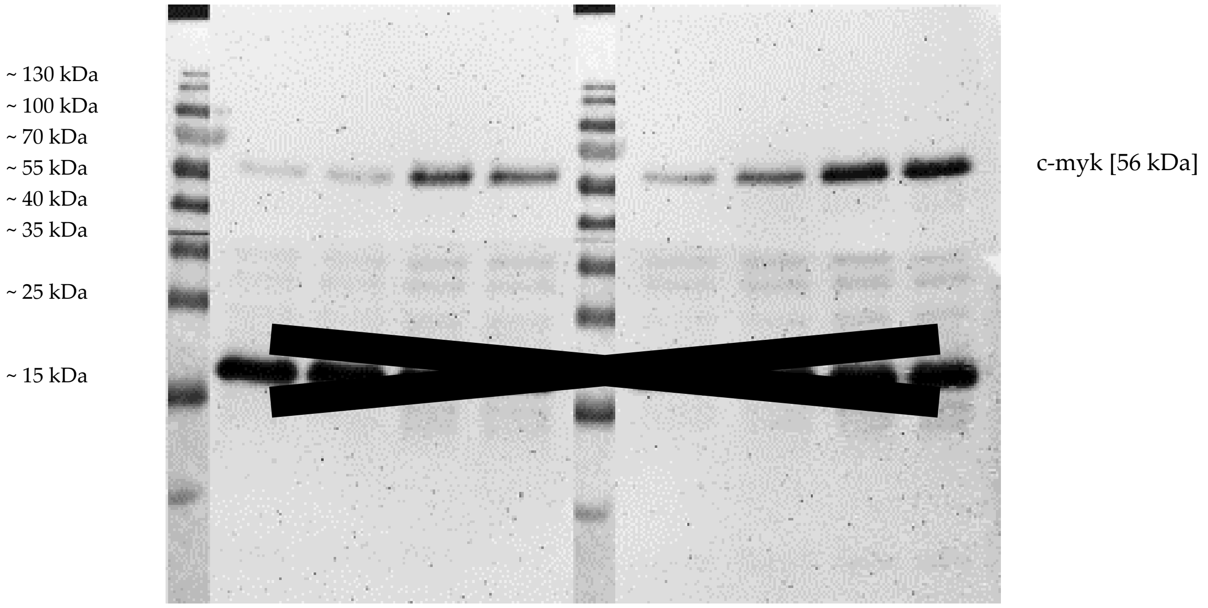
~ 130 kDa
~ 100 kDa
~ 70 kDa
~ 55 kDa
~ 40 kDa
~ 35 kDa
~ 25 kDa
~ 15 kDa
c-myk [56 kDa]

c)

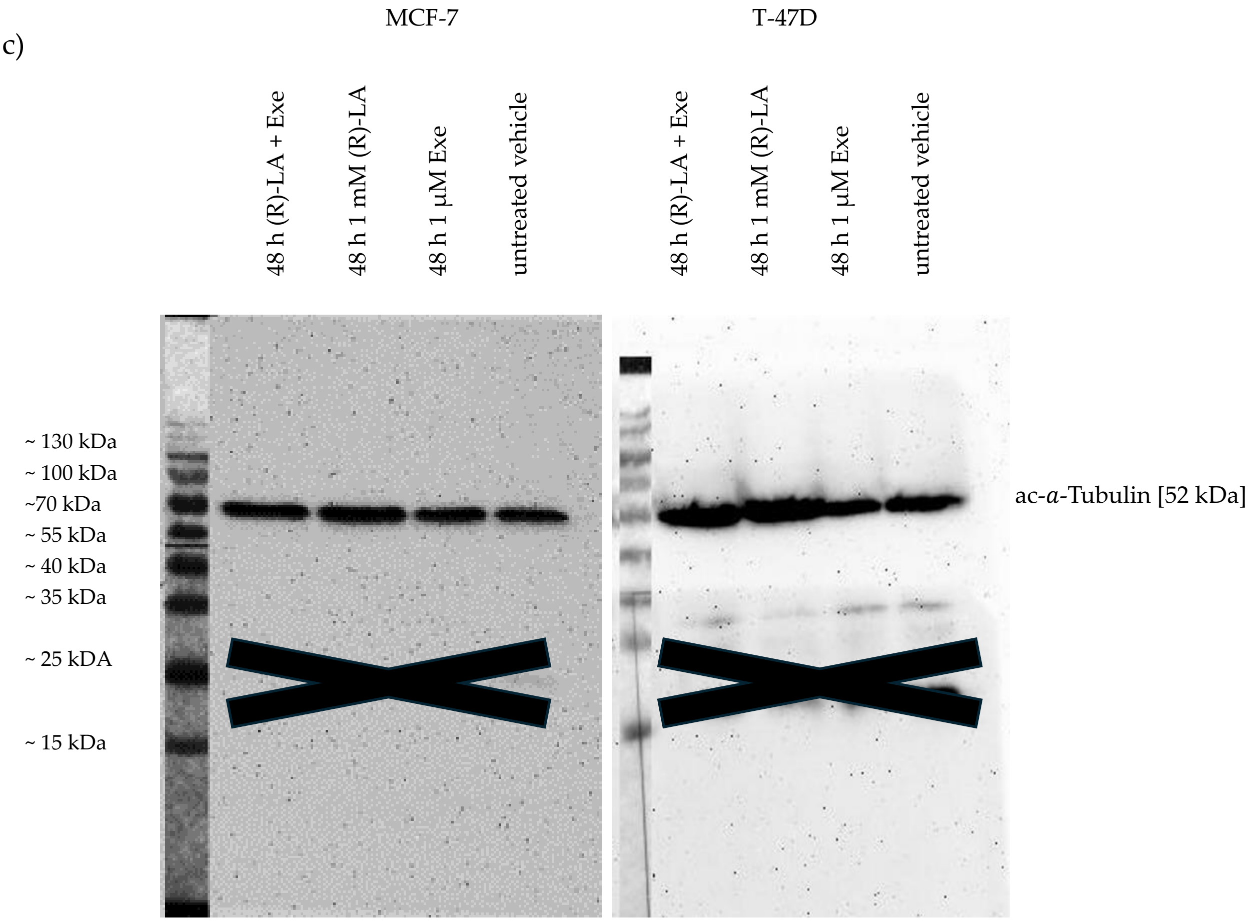
MCF-7
T-47D
48 h (R)-LA + Exe
48 h 1 mM (R)-LA
48 h 1 µM Exe
untreated vehicle
48 h (R)-LA + Exe
48 h 1 mM (R)-LA
48 h 1 µM Exe
untreated vehicle
~ 130 kDa
~ 100 kDa
~70 kDa
~ 55 kDa
~ 40 kDa
~ 35 kDa
~ 25 kDA
~ 15 kDa
ac-α-Tubulin [52 kDa]

d)

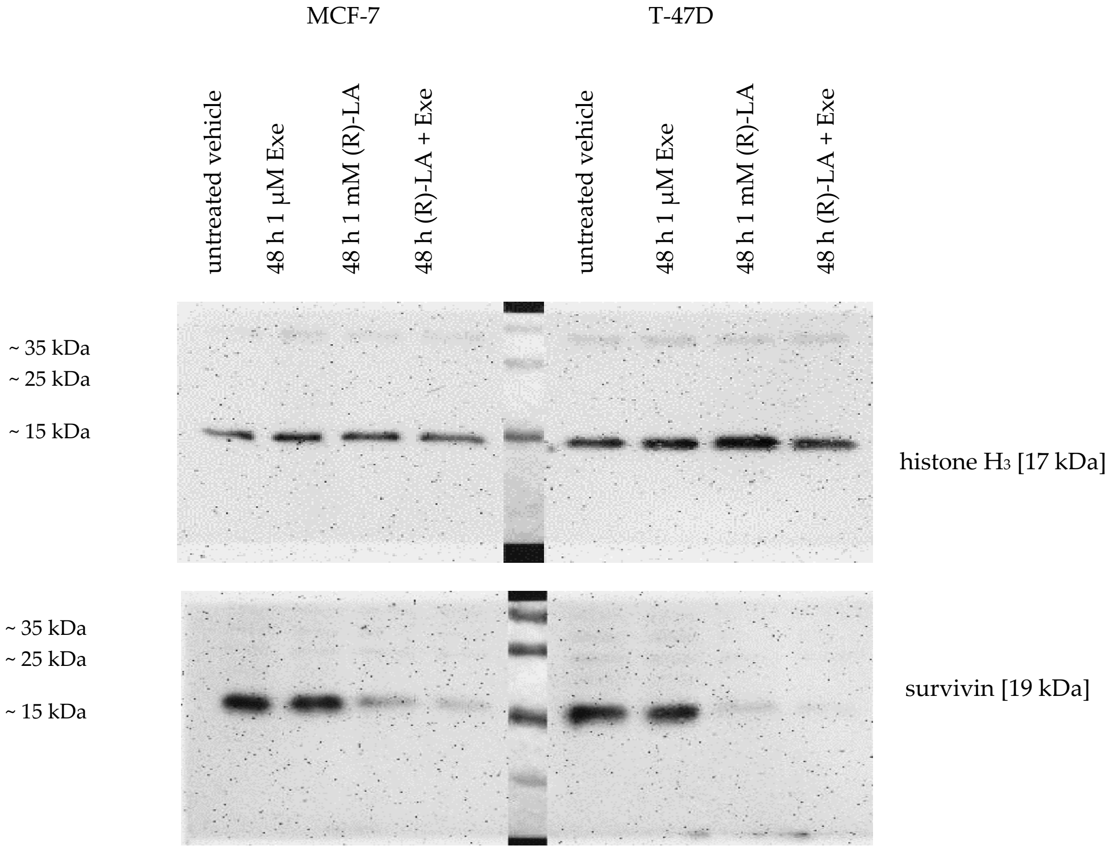
MCF-7
T-47D
untreated vehicle
48 h 1 µM Exe
48 h 1 mM (R)-LA
48 h (R)-LA + Exe
untreated vehicle
48 h 1 µM Exe
48 h 1 mM (R)-LA
48 h (R)-LA + Exe
~ 35 kDa
~ 25 kDa
~ 15 kDa
histone H3 [17 kDa]
~ 35 kDa
~ 25 kDa
~ 15 kDa
survivin [19 kDa]

e)

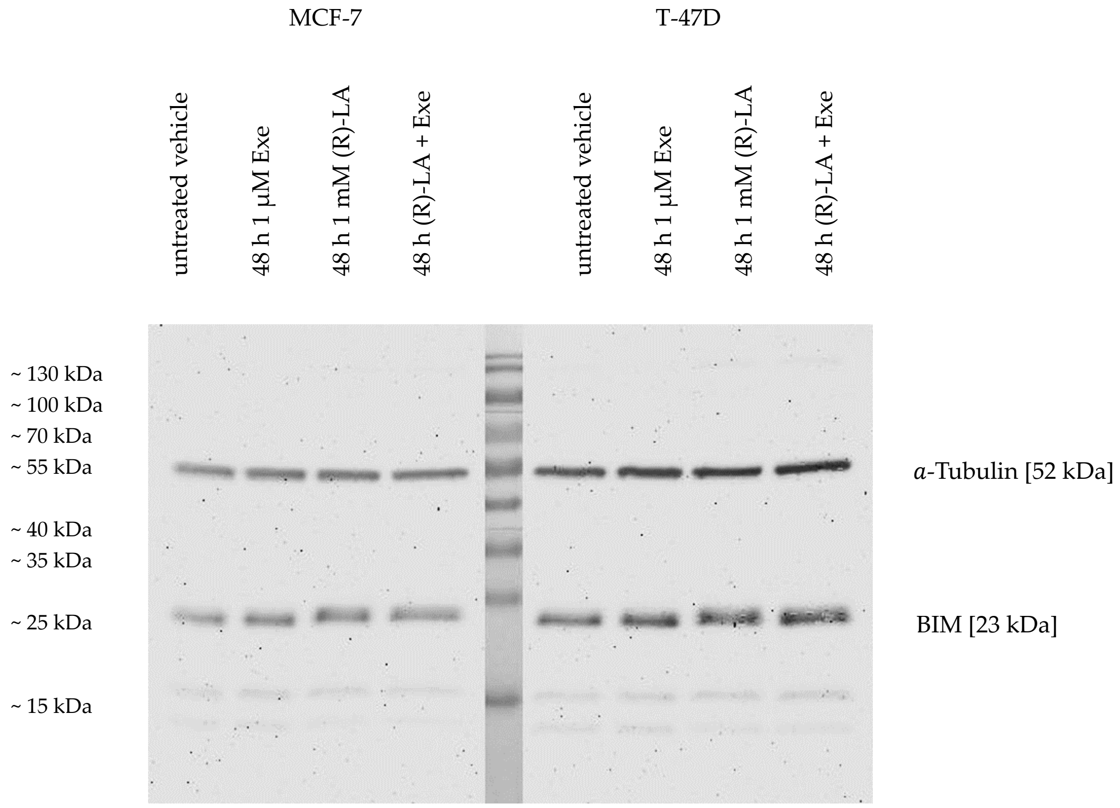
MCF-7
T-47D
untreated vehicle
48 h 1 µM Exe
48 h 1 mM (R)-LA
48 h (R)-LA + Exe
untreated vehicle
48 h 1 µM Exe
48 h 1 mM (R)-LA
48 h (R)-LA + Exe
~ 130 kDa
~ 100 kDa
~ 70 kDa
~ 55 kDa
~ 40 kDa
~ 35 kDa
~ 25 kDa
~ 15 kDa
α-Tubulin [52 kDa]
BIM [23 kDa]

f)

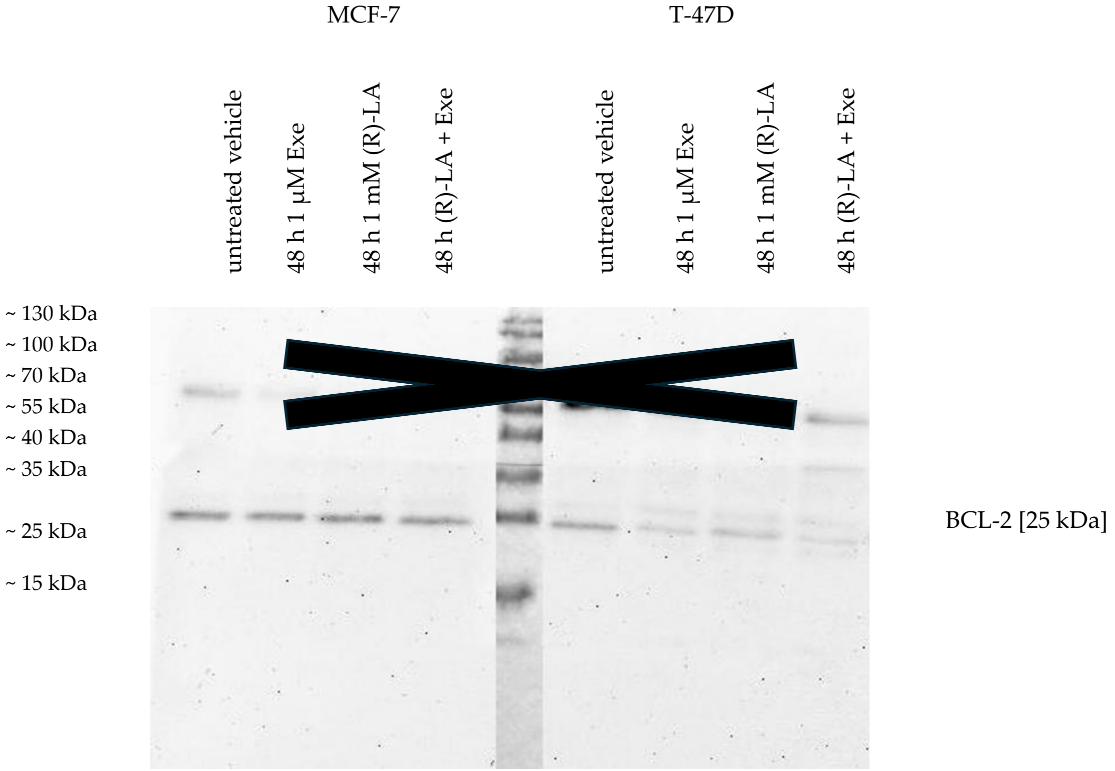
MCF-7
T-47D
untreated vehicle
48 h 1 µM Exe
48 h 1 mM (R)-LA
48 h (R)-LA + Exe
untreated vehicle
48 h 1 µM Exe
48 h 1 mM (R)-LA
48 h (R)-LA + Exe
~ 130 kDa
~ 100 kDa
~ 70 kDa
~ 55 kDa
~ 40 kDa
~ 35 kDa
~ 25 kDa
~ 15 kDa
BCL-2 [25 kDa]

g)

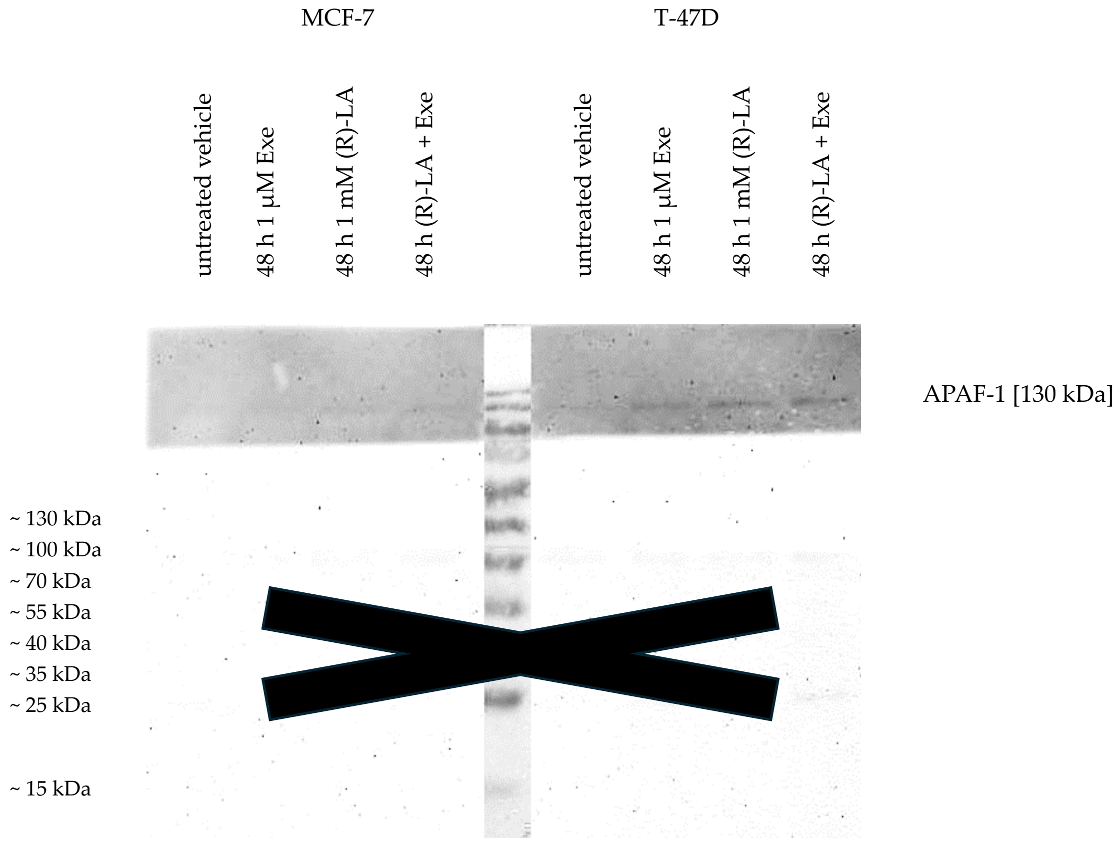
MCF-7
T-47D
untreated vehicle
48 h 1 μM Exe
48 h 1 mM (R)-LA
48 h (R)-LA + Exe
untreated vehicle
48 h 1 μM Exe
48 h 1 mM (R)-LA
48 h (R)-LA + Exe
APAF-1 [130 kDa]
~ 130 kDa
~ 100 kDa
~ 70 kDa
~ 55 kDa
~ 40 kDa
~ 35 kDa
~ 25 kDa
~ 15 kDa

h)

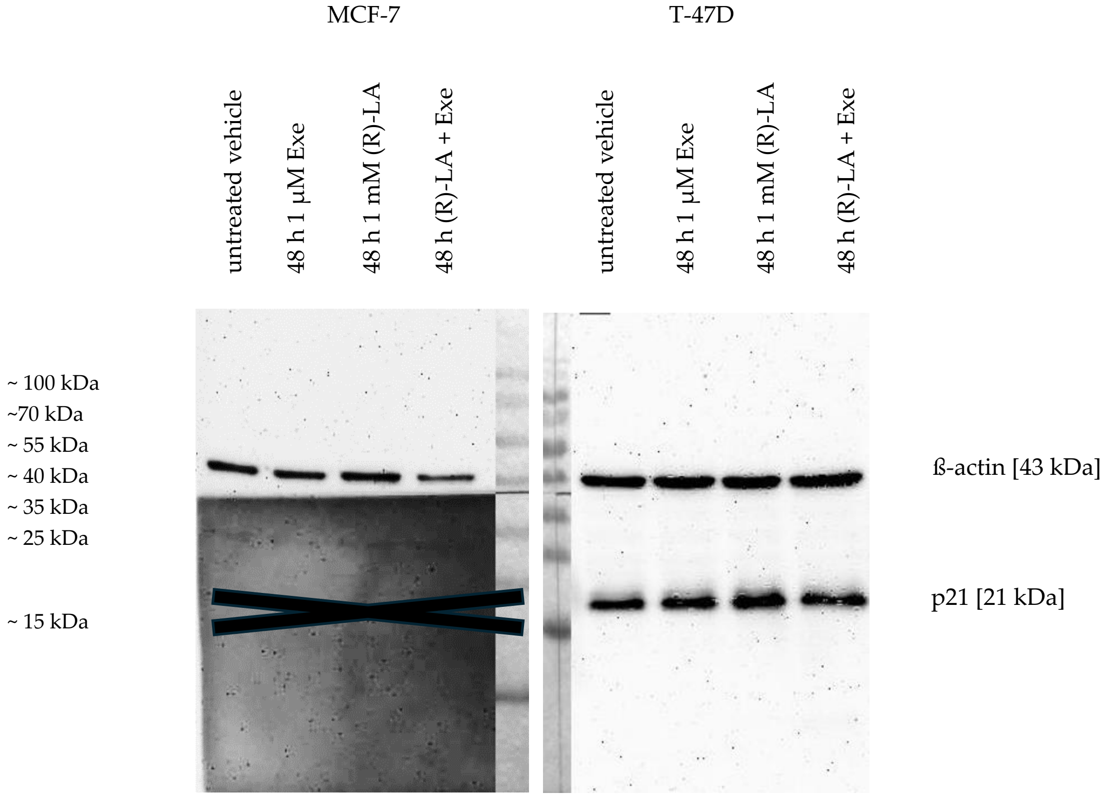
MCF-7
T-47D
untreated vehicle
48 h 1 μM Exe
48 h 1 mM (R)-LA
48 h (R)-LA + Exe
untreated vehicle
48 h 1 μM Exe
48 h 1 mM (R)-LA
48 h (R)-LA + Exe
~ 100 kDa
~70 kDa
~ 55 kDa
~ 40 kDa
~ 35 kDa
~ 25 kDa
~ 15 kDa
ß-actin [43 kDa]
p21 [21 kDa]

i)

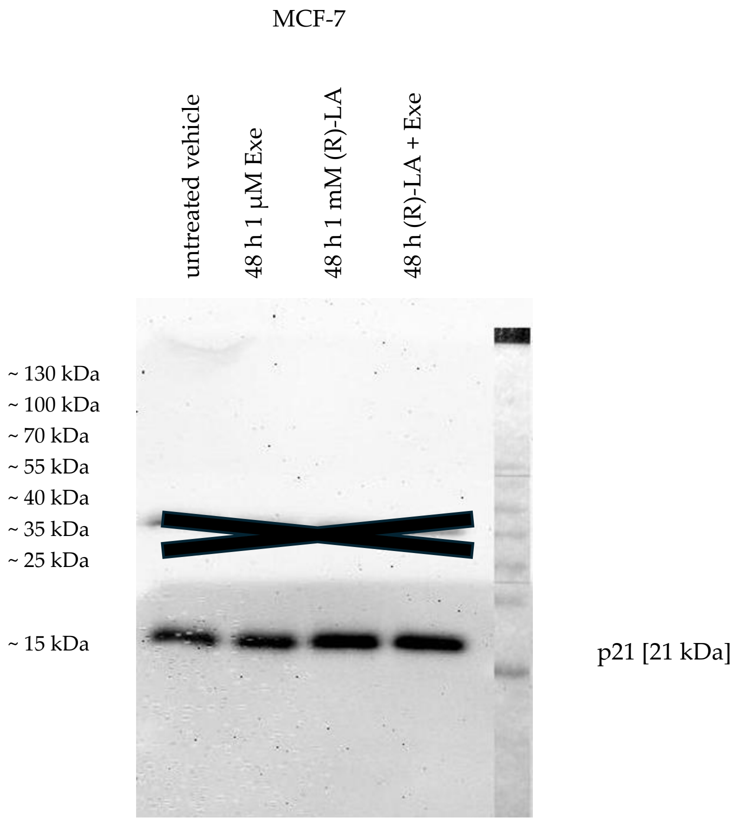

Figure S2. Effects of *(R)-α*-lipoic acid and exemestane on protein expression levels of apoptosis- and proliferation-related proteins and on acetylation levels of *a*-tubulin and histone H3 in MCF-7 and T47D cells. Shown are representative immunoblots of FOXO1 and ac-histone H3 (a), c-myk (b), ac-a-tubulin (c), histon H3 and survivin (d), a-tubulin and BIM (e), BCL-2 (f), APAF-1 (g), ß-actin and p21 in T47D (h) and p21 in MCF-7 (i). Cells were treated for 48h with the given compounds and concentrations. Protein weight marker is indicated next to the corresponding blot. Exe = exemestane. (R)-LA = *(R)-α*-lipoic acid.

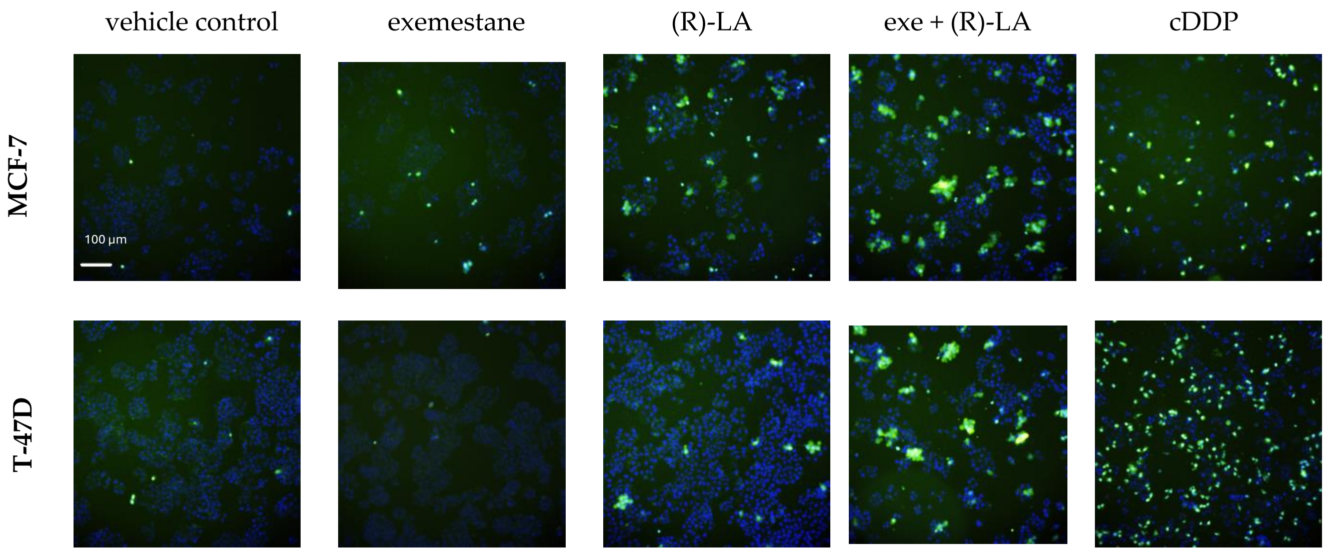

Figure S3a. Caspase 3/7-activation in MCF-7 and T47D cells. Displayed are representative fluorescent images for each treatment (10x magnification) from a set of 2 experiments. Cells were incubated with 1 µM exemestane, 0.5 mM *(R)-α*-lipoic acid, the combination and 100 µM cisplatin for 24 h. Hoechst 33342 was used to stain cell nuclei (blue) and CellEvent Caspase-3/7 green detection reagent for green-colored activated caspase 3/7. Scale bar in upper left image is 100 µm and applies to all images. Exe = exemestane. (R)-LA = *(R)-α*-lipoic acid. cDDP = cisplatin.

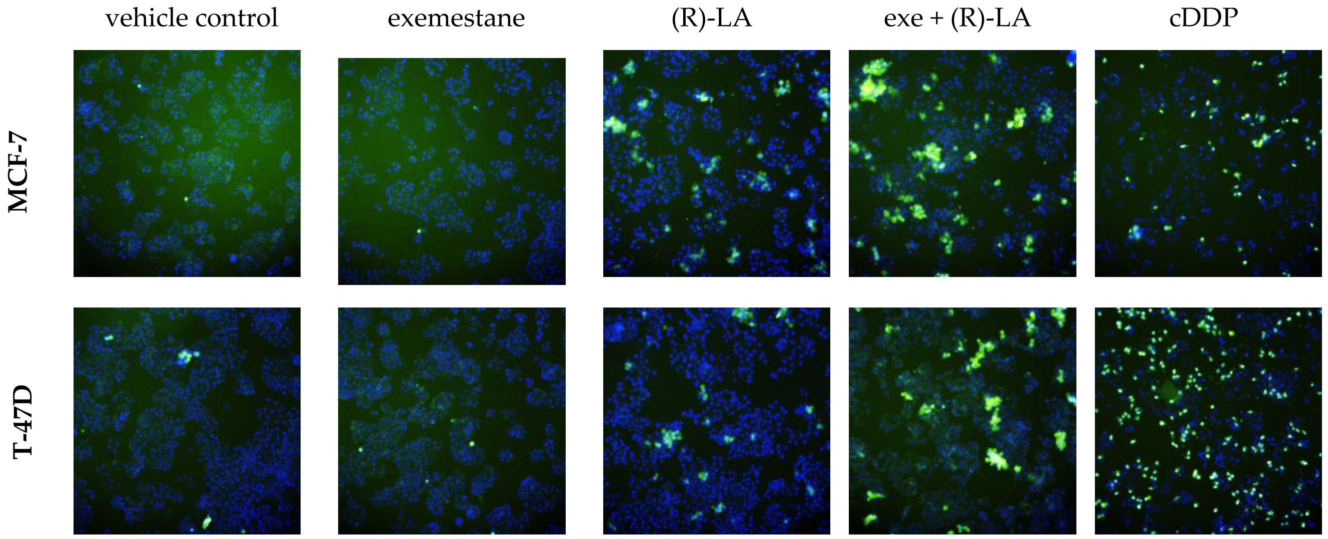

Figure S3b. Caspase 3/7-activation in MCF-7 and T47D cells. Displayed are representative fluorescent imaging pictures for each treatment (10x magnification) from a set of 2 experiments. Cells were incubated with 1 µM exemestane, 1 mM *(R)-α*-lipoic acid, the combination and 100 µM cisplatin for 24h. Hoechst 33342 was used to stain cell nuclei (blue) and CellEvent Caspase-3/7 green detection reagent for green-colored activated caspase 3/7. Scale bar see Figure 12. Exe = exemestane. (R)-LA = *(R)-α*-lipoic acid. cDDP = cisplatin.

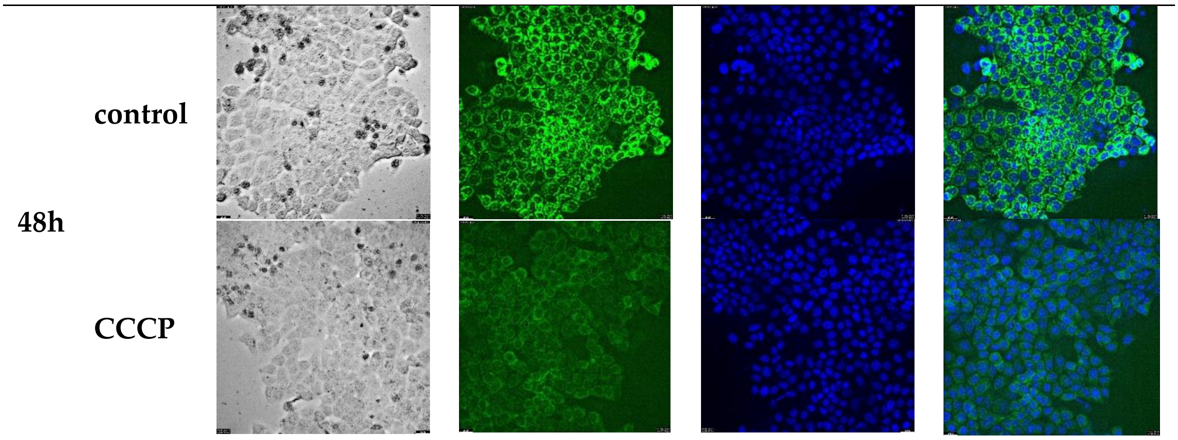

Figure S4. Mitochondrial potential in MCF-7 cells. Displayed are representative fluorescence images (out of two independent experiments) of untreated control and 20 μM CCCP. First column shows a brightfield microscope image of cells. Second column shows mitochondrial potential analyzed by TMRE (green) and third column nuclei stained by Hoechst 33342 (blue). Fourth column: merged mitochondrial potential and nuclei. 90 minutes before staining control wells were incubated with 20 μM CCCP. Magnification: 40x. Scale bar is shown in the upper left image in Figure 16 and applies to all images. (R)-LA = *(R)*-α-lipoic acid.

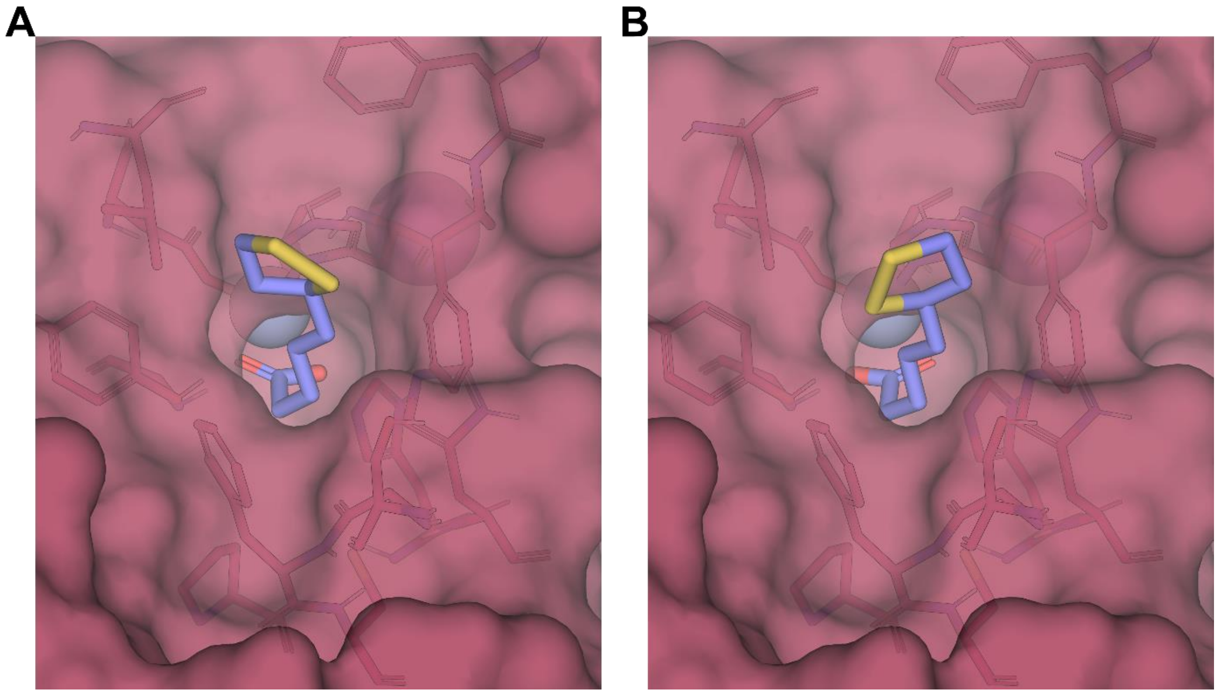

Figure S5. Surface view of predicted binding poses of *(R)*-α-lipoic acid (A) and *(S)*-α-lipoic acid (B). The best docking pose of each structure (blue) out of 100 docking poses in human HDAC 6 (dark pink) is shown. Residues in close vicinity to the bound α-lipoic acid are shown as sticks.

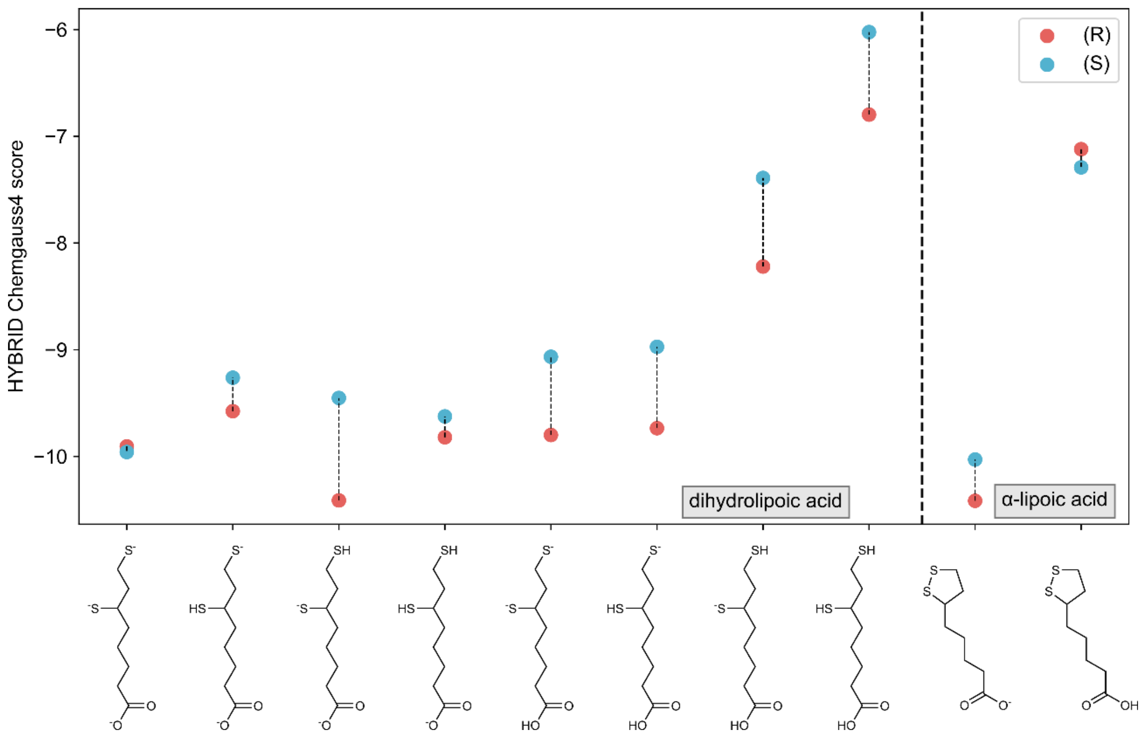

Figure S6. Summary of docking scores for all protonation states and enantiomers of dihydro-lipoic acid and *α*-lipoic acid. (*R*) forms are shown with red dots and (*S*) forms with blue dots.
